# The Role of p66Shc in Cancer: Molecular Mechanisms and Therapeutic Implications

**DOI:** 10.1111/jcmm.70737

**Published:** 2025-07-21

**Authors:** Davood Zaeifi, Khadijeh Jamialahmadi, Gholamreza Karimi

**Affiliations:** ^1^ Department of Medical Biotechnology and Nanotechnology, Faculty of Medicine Mashhad University of Medical Sciences Mashhad Iran; ^2^ Biotechnology Research Center, Pharmaceutical Technology Institute, Faculty of Medicine Mashhad University of Medical Sciences Mashhad Iran; ^3^ Pharmaceutical Research Center, Institute of Pharmaceutical Technology, Faculty of Pharmacy Mashhad University of Medical Sciences Mashhad Iran; ^4^ Department of Pharmacodynamics and Toxicology School of Pharmacy Mashhad Iran

**Keywords:** cancer, p66Shc, reactive oxygen species, signalling pathway

## Abstract

p66Shc is a redox‐sensitive and pro‐apoptotic adaptor protein that regulates oxidative stress and mitochondrial apoptosis. It is the largest of three isoforms encoded by the proto‐oncogene ShcA (Src collagen homologue A). Members of the ShcA family are capable of recruiting various signalling molecules and are involved in several cellular pathways, including proliferation, growth and survival. Increasing evidence highlights the p66Shc role in various tumourigenic processes, such as cell expansion, progression, metastasis and metabolic reprogramming. This review summarises current knowledge on the role of p66Shc in cancer, explains the molecular mechanisms underlying the effects of this protein, and considers therapeutic prospects aimed at targeting it. Emerging therapeutic strategies, including small‐molecule inhibitors and gene‐editing approaches, are discussed alongside challenges in clinical translation.

**Abbreviations**
ADT: androgen deprivation therapy
AR: androgen receptor
ASOs: antisense oligonucleotides
BC: breast cancer
BCL‐2: B‐cell lymphoma 2
CLL: chronic lymphocytic leukaemia
co‐IP: co‐immunoprecipitation
CRC: colorectal cancer
CSCs: cancer stem cells
Cytc: cytochrome C
DHT: dihydrotestosterone
DN: diabetic nephropathy
DZ: *Dioscorea zingiberensis*
EMT: epithelial‐mesenchymal transition
ERK: extracellular signal‐regulated kinases
GPX: glutathione peroxidase
HCC: hepatocellular carcinoma
HER2: ErbB2 (human epidermal growth factor receptor 2)
IKK: Iκb kinas
MAPK/ERK: mitogen‐activated protein kinase/extracellular signal‐regulated kinase
MEK: mitogen‐activated protein/extracellular signal‐regulated kinase kinase
MMP: matrix metalloproteinase
MS: mass spectrometry
NAFLD: non‐alcoholic fatty liver disease
NSCLC: non‐small cell lung carcinoma
OC: ovarian cancer
PC: pancreatic cancer
PCa: prostate cancer
PH domain: pleckstrin homology domain
PI: phosphatidylinositol
PI3K/Akt: phosphatidylinositol 3‐kinase/protein kinase B
PTB: phosphotyrosine binding
PTMs: post‐translational modifications
Rb: retinoblastoma
ROS: reactive oxygen species
RTKs: receptor tyrosine kinases
SH2: Src homology 2
ShcA: Src collagen homologue A
SNTA1: Alpha‐1‐syntrophin
SOD: superoxide dismutase
TKIs: tyrosine kinase inhibitors
TME: tumour microenvironment
TNBC: triple‐negative breast cancer

## 1 Introduction

**FIGURE 1:**
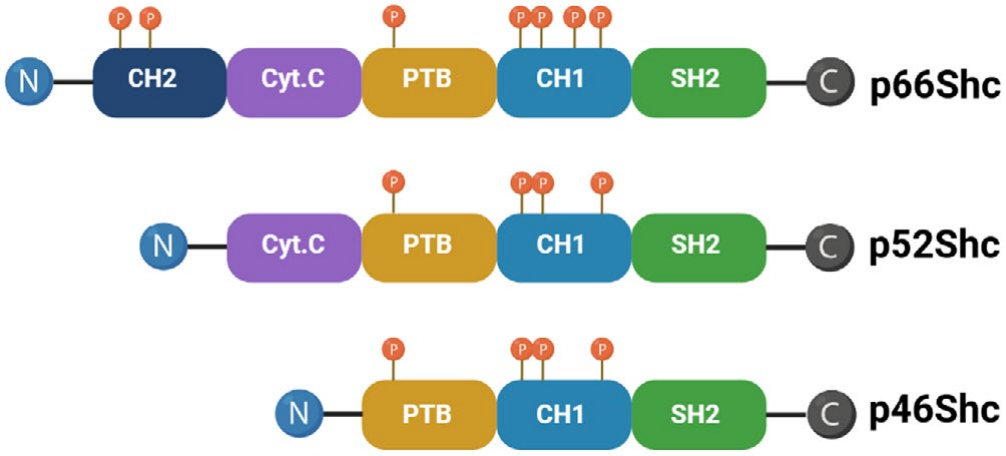
Schematic structure of Shc proteins: P66Shc, p52Shc and p46Shc isoforms. Each isoform is conserved by distinct functional domains, including PTB, CH1 and SH2; the cytochrome C binding domain is found in p52Shc and p66Shc; however, p66Shc also represents CH2. The CH1 domain is phosphorylated in response to activated receptor tyrosine kinases, which promote mitogenic responses. In contrast, phosphorylation and acetylation of the CH2 domain trigger oxidative responses. Phosphorylation sites represented (P). Created with BioRender.com [1].

p66Shc was identified in the early 1990s and is the largest isoform of the ShcA family (alongside p52Shc and p46Shc) and uniquely harbours a CH2 domain critical for redox activity (Figure 1). The Shc family of adaptor proteins is involved in different cell signalling pathways to mediate cellular responses to different types of stimuli. p66Shc is mainly involved in the cellular response to oxidative stress, unlike the other members of this protein family, which regulate apoptosis. This protein also exhibits a dual role in cancer, promoting either apoptosis or cell survival based on cellular outcomes, growth factors and environmental stressors [1, 2].

Reactive oxygen species (ROS) imbalance, characterised by excessive ROS production relative to cellular antioxidant capacity, is a critical driver of tumourigenesis. High ROS levels induce DNA damage, genomic instability and aberrant signalling, promoting cancer initiation and progression. The p66Shc protein, a key redox‐sensitive adaptor, is activated by elevated intracellular ROS, which triggers its translocation to the mitochondria, enhances ROS production by oxidising cytochrome C (Cytc), and initiates the mitochondrial apoptosis pathway through the release of pro‐apoptotic factors, such as Cytc and SMAC/DIABLO [1, 2]. In cancers like breast cancer (BC), high p66Shc expression enhances chemotherapy sensitivity by promoting reactive oxygen species (ROS)‐mediated apoptosis, yet in other contexts, it drives tumour survival through pathways like PI3K/Akt, modulated by tumour type and microenvironment. For instance, p66Shc can activate the phosphatidylinositol 3‐kinase/protein kinase B (PI3K/Akt) pathway, a critical regulator of cell proliferation and survival, by facilitating the recruitment of signalling molecules to receptor tyrosine kinases (RTKs). This activation promotes anti‐apoptotic signals, enhances glucose metabolism and contributes to tumour progression, underscoring the complexity of p66Shc's role in cancer biology [3, 4].

Encoded by the SHC1 gene, p66Shc contains distinct domains (SH2, CH1, PTB, CH2) that enable it to mediate RTKs signalling and regulate redox balance, distinguishing it from p52Shc and p46Shc isoforms. Since the CH2 domain is specific to p66Shc, this domain is vital for that protein's redox activity. Besides acetylation, phosphorylation causes a mitochondrial translocation along with ROS production. This redox function links p66Shc with oxidative stress responses, altering mitochondrial signalling to promote apoptosis when Cytc is released or, under certain conditions, facilitating cell survival through anti‐apoptotic pathways. These structural features enable p66Shc to function as a flexible adapter protein, linking mitogenic signals to oxidative stress reactions in cancer biology [1, 5]. p66Shc acts as more of a scaffold inside protein complex assembly for propagating signals away from phosphorylated receptors and into effector cascades. These signals influence cellular metabolism, as well as mitogenesis and programmed cell death. Studies indicate that p66Shc exerts a dual role in cell growth and apoptosis [5], particularly in cardiovascular pathology, acting as a rheostat in the regulation of apoptosis mediated by interactions with Cytc [6]. It has been linked to endothelial dysfunction related to hyperlipidemia, diabetes and ageing [7], with its expression rising in human fibroblasts [8] as people age and in age‐related diseases like prostate cancer (PCa) [5, 8]. Additionally, p66Shc is involved in the proliferation and metastasis of androgen‐regulated PCa cells through redox signalling pathways [8].

Although p66Shc's role in tumorigenesis is increasingly recognised, its interactions with oncogenes, tumour suppressors, post‐translational modifications and therapeutic potential remain underexplored [9]. Therefore, this review aimed to integrate current findings relating to the role of p66Shc in the biology of malignancies, explain the molecular mechanisms involved and discuss possible therapeutic approaches targeting this protein, which provide valuable information on possible novel approaches to cancer treatment in general and specifically to tumours that become resistant to conventional treatments.

## 2 Molecular Mechanisms of p66Shc in Cancer

Studies on humans have revealed that p66Shc has been involved as a significant factor in the development of diabetes, cardiovascular disease, neurodegeneration, inflammation, obesity, ageing and certain cancers.

### 2.1 p66Shc and Signal Transduction Pathways

**FIGURE 2:**
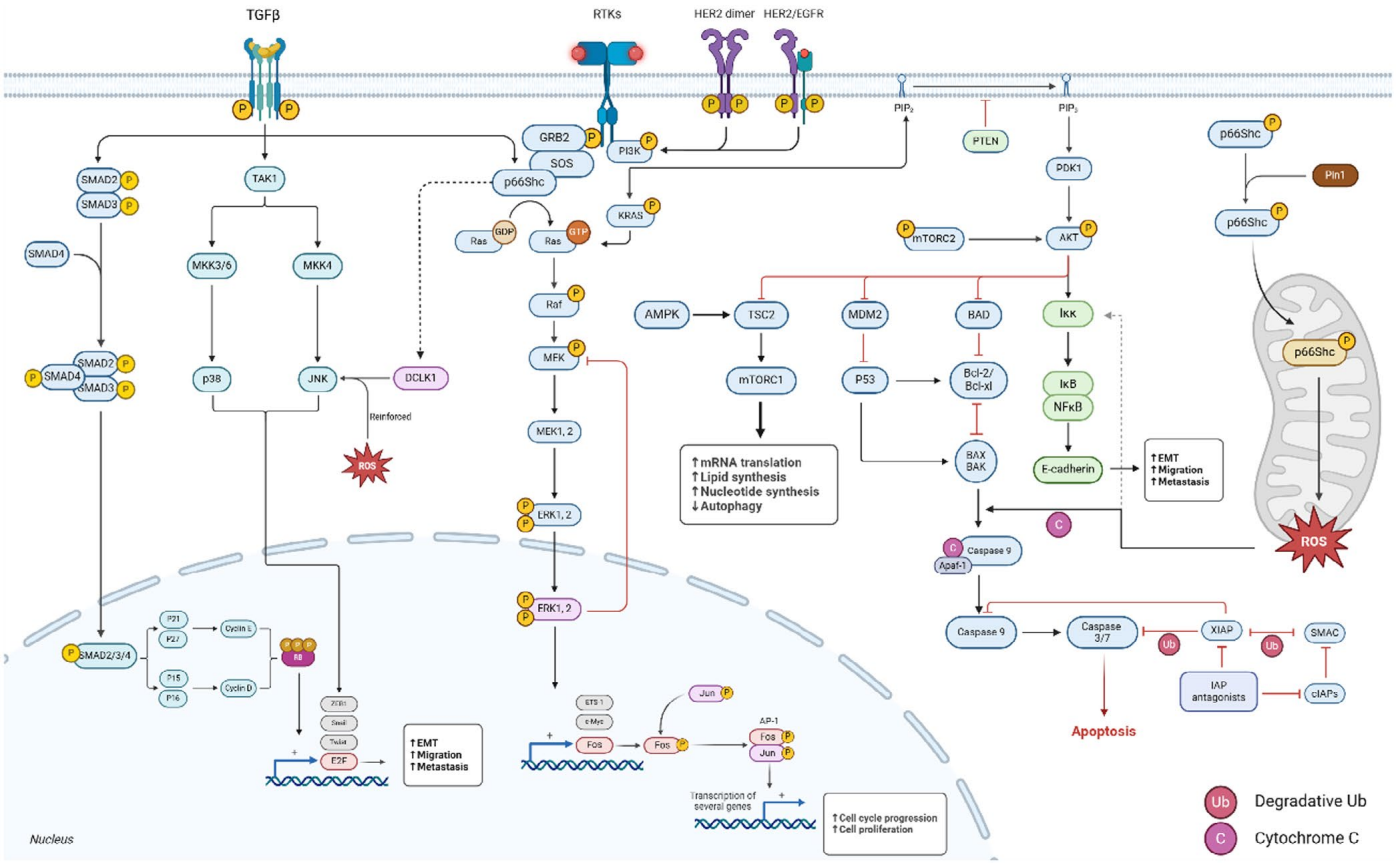
Pathway interactions involving p66Shc: This diagram represents key signalling pathways, including the PI3K/Akt pathway, MAPK/ERK pathway and NF‐κB pathway, which are influenced by p66Shc and their interplay with apoptotic mechanisms. Upon activation by RTKs and TGF‐β, p66Shc facilitates critical phosphorylation events that drive cellular processes, including mRNA translation, lipid synthesis, cell proliferation and EMT. Additionally, it reveals how p66Shc plays as a hub connecting these pathways, promoting tumoral migration and metastasis while modulating apoptotic signals through interactions with BCL‐2 family proteins and Caspases. Additionally, the crosstalk between these pathways highlights the complexity of tumour biology. Created with BioRender.com [3, 4, 10].

The p66Shc becomes activated by growth factors (such as EGF and PDGF) and stress signals (including oxidative stress, hypoxia and nutrient deprivation) to initiate signalling cascades. The involvement of p66Shc in various cancer signalling systems, including cell survival and proliferation through PI3K/Akt, MAPK and mTOR, apoptosis regulation, inflammation and immune response by NF‐κB, development and differentiation with TGF‐β, cell cycle regulation and stress response represents a central role in oncogenic signalling networks [4]. The involvement of p66Shc in integrin signalling, facilitated by its interaction with Rac1, a small GTPase, suggests that it may contribute to enhanced capability of cancer cell migration, which supports metastasis [4]. Its function in metabolic regulation and tumourigenesis depends on the connection between oxidative stress responses and wider signalling pathways such as the AMPK pathway (Figure 2).

p66Shc serves as a versatile adaptor protein in cancer, mediating RTKs and stress‐induced signalling to regulate pathways like PI3K/Akt, MAPK/ERK, NF‐κB and TGF‐β. Through ROS generation and mitochondrial interactions, it influences cell survival, proliferation, apoptosis and epithelial–mesenchymal transition (EMT), with effects varying by tumour type and microenvironment.

#### 2.1.1 PI3K/Akt Pathway

p66Shc dynamically regulates the PI3K/Akt pathway, a central driver of cancer cell survival and chemoresistance, via its PTB domain‐mediated recruitment to RTKs [10, 11]. Model systems of p66Shc knock‐out show that altering the Shc proteins in cellular content could selectively influence the activation of PI3K and Akt, suggesting that p66Shc can act as a modulatory switch in this pathway [10, 12]. Such a proposition suggests that the PI3K/Akt pathway plays a central role in the onset of tumorigenesis, and its abnormal activation is mainly responsible for the malignant characteristics of cancers, including breast and colorectal origin [10, 13].

Different growth factors and oncogenic signals induce the PI3K/Akt pathway activation through binding to their respective RTKs, which leads to Akt phosphorylation, receptor dimerisation and autophosphorylation, thereby creating docking sites for downstream signalling molecules [14]. Upon activation, p66Shc engages the plasma membrane using the phosphotyrosine binding (PTB) domain and binds to phosphorylated tyrosine residues on activated RTKs; p66Shc activates RAS by GDP/GTP exchange [15]. In the following, PI3K becomes activated and phosphorylates phosphatidylinositol (PI) at the 3‐position of the inositol ring, converts it into PIP3, and prepares a docking site for engaging Akt through its pleckstrin homology (PH) domain, where it is phosphorylated and activated by PDK1 and mTORC2 [16].

The Akt initiates a cascade of downstream signalling once activated, including (I) the inhibition of apoptosis by phosphorylation and inactivation of pro‐apoptotic factors such as Bad and FoxO transcription factors [17], (II) promoting cell cycle progression by phosphorylating and inhibiting cyclin‐dependent kinase inhibitors (such as p21 and p27), allowing for the transition from G1 to S phase [18], and (III) enhancing glucose uptake and metabolism by promoting the translocation of GLUT4 to the cell membrane and stimulating glycolysis [19]. Notably, p66Shc's Ser36 phosphorylation by PKCβ enhances PI3K/Akt activation, linking oxidative stress to pro‐survival signalling [20]. All these event series underscore their role in cancer progression.

The p66Shc appears to play a crucial role in cancer cells by mediating chemotherapy resistance, a significant barrier to effective cancer treatment [21]. Evidence shows that p66Shc promotes chemoresistance through the PI3K/Akt pathway, which conveys anti‐apoptotic signals that protect cancer cells from chemotherapeutic drugs [22]; therefore, targeting p66Shc or its downstream effects could be a sensible strategy to improve treatment outcomes by overcoming such resistance mechanisms.

#### 2.1.2 MAPK/ERK Pathway

The classical MAPK pathway, specifically the RAF/MEK/ERK cascade, is one of the most commonly disrupted pathways in cancer, leading to increased cell growth and decreased apoptosis [23], which adds another layer of complexity by functioning as a negative regulator of the RAS/MAPK pathway. The p66Shc may apply a tumour‐suppressing effect by inhibiting the RAS signalling pathway; however, its overexpression in certain cancers is associated with a more aggressive tumour phenotype [1].

RAS activation occurs similarly to the PI3K/Akt pathway. The activated protein interacts with and activates RAF, a serine/threonine kinase. This interaction causes RAF to undergo a conformational change, enhancing its kinase activity to phosphorylate and activate mitogen‐activated protein/extracellular signal‐regulated kinase kinase (MEK), with BRAF being particularly significant in oncogenesis (Table 1) [37].

**TABLE 1:** The p66Shc interactions with oncogenes and tumour suppressors, the prognostic significance of p66Shc expression and its role in different cancer types.

Oncogene/Tumour suppressor	Nature of interaction	Implications for cancer progression	p66Shc expression level	Cancer type	Associated signalling pathways	References
c‐MYC	Activation	Promotes cell proliferation and survival	Upregulated	Various (e.g., breast, lung)	PI3K/Akt, MAPK/ERK	[24]
p53	Inhibition	Loss of tumour suppressive function in cancer	Downregulated	Various (e.g., colorectal, breast)	p53 signalling, apoptosis	[25]
RAS	Activation	Enhances oncogenic signalling pathways	Upregulated	Pancreatic, colorectal	RAS/MAPK, PI3K/Akt	[26, 27]
PTEN	Inhibition	Disruption of tumour suppressive effects	Downregulated	Prostate, glioblastoma	PI3K/Akt	[28, 29]
BAX/BCL‐2	Activation	Induces apoptosis in response to stress	Upregulated	Various (e.g., leukaemia)	Apoptosis signalling	[30]
Mortalin	Activation	Promotes cell survival and proliferation	Upregulated	Lung, breast	HSF1, mitochondrial signalling	[31, 32]
Pin1	Activation	Regulates protein stability and function	Upregulated	Breast, prostate	Cell cycle regulation	[33, 34]
Rb	Inhibition	Loss of cell cycle control	Downregulated	Retinoblastoma, small cell lung cancer	Rb/E2F pathway	[35]
SIRT1	Activation	Modulates metabolism and stress response	Upregulated	Various (e.g., breast, prostate)	SIRT1/AMPK, metabolic pathways	[36]

Activated MEK phosphorylates and activates ERK1 and ERK2 (extracellular signal‐regulated kinases), specifically on key residues (Thr202 and Tyr204) crucial for full activation. Active ERK then translocates to the nucleus and regulates transcription factors [38, 39, 40].

The activated ERK initiates a cascade of downstream signalling that includes (I) promoting cell cycle progression by activation of immediate‐early genes (like c‐Fos, c‐Jun, c‐MYC and Ets‐1) and essential transcription factors [24], (II) enhancing cell viability by phosphorylation of anti‐apoptotic proteins (e.g., BCL‐2 and p53) and promoting the activity of transcription factors that counteract pro‐apoptotic signals [25], (III) influencing cytoskeletal dynamics through phosphorylation of substrates such as VASP and cofilin, facilitating cellular movement and morphological changes relevant for migration and metastasis [41].

Research has demonstrated that p66Shc can negatively regulate the RAS/MAPK/Fos pathway by blocking essential Grb2‐Sos complexes for RAS activation [1]. The pathway induces cell division and differentiation, and its deregulation may cause tumour development and progression. p66Shc shows additional complexity by participating in both pro‐apoptotic and pro‐survival signalling pathways in tumour biology.

Additionally, p66Shc regulates oxidative stress, which is closely linked to MAPK signalling. Oxidative stress typically activates several MAPK pathways, including ERK, p38‐MAPK and all common traits in cancer cells [38]. The p66Shc can be phosphorylated in response to oxidative stress, leading to an increased production of ROS [42], which activates the MAPK pathways in a positive feedback loop that can intensify oxidative stress and potentially contribute to tumourigenesis. For example, p66Shc has been shown to mediate oxidative stress induced by high glucose and angiotensin II through mitochondrial‐dependent apoptotic pathways [43]. Understanding the interactions between p66Shc, oxidative stress and MAPK signalling is essential for elucidating its role in cancer, as elevated ROS levels are often associated with tumour progression and metastasis [44].

#### 2.1.3 NF‐κB Pathway

The NF‐κB pathway activation can be triggered by multiple stimuli, including cytokines (like TNF‐α), growth factors and cellular stress signals. These triggers can activate p66Shc and lead to translocation from the cytoplasm to the plasma membrane. The role of p66Shc in modulating NF‐κB activity in different types of cancers highlights its relevance as a negative regulator of this crucial signalling pathway [45, 46].

The p66Shc modulates the activation of the IκB kinase (IKK) complex that occurs through the generation of ROS or other intermediates that activate IKK. Then, the IKK complex phosphorylates IκB proteins (inhibitors of NF‐κB), leading to their ubiquitination and subsequent degradation by the proteasome. The degradation of IκB proteins releases held NF‐κB dimers (commonly p65/RelA and p50 subunits) that are in the cytoplasm in an inactive form. Once free, these dimers translocate to the nucleus [47, 48]. The activated NF‐κB dimers bind to specific DNA response elements and promote the transcription of target genes, including cytokines (e.g., IL‐1β, IL‐6 and TNF‐α), anti‐apoptotic proteins (e.g., BCL‐2 and cIAPs) and cell cycle regulators (e.g., cyclin D1 and c‐Myc) involved in inflammation, immune response, cell survival and proliferation (Table 1) [45, 49, 50].

The activated NF‐κB can generate feedback signals that regulate the activity of p66Shc and other components of the signalling pathway by transcribing superoxide dismutase (SOD1 and SOD2), glutathione peroxidase (GPX), NQO1 and catalase genes, which play crucial roles in modulating oxidative stress levels within the cell. By increasing the expression of these antioxidant enzymes, NF‐κB can reduce the accumulation of ROS produced during cellular stress and lead to decreased activation of p66Shc [51].

It is reported that p66Shc deficiency is associated with an increased NF‐κB activity, which leads to an upregulation of chemokine receptors (PD‐L1 expression) [52] through a ROS‐dependent mechanism [53]. This indicates that the pro‐oxidant activity of p66Shc is essential for maintaining NF‐κB signalling homeostasis [52], as elevated levels of ROS can inhibit NF‐κB transcriptional activity [54]. Ingersoll et al. underlined the role of p66Shc in facilitating the migratory drug‐resistant cancer cells' function through ROS generation, suggesting that p66Shc activity is also related to tumour aggressiveness and therapeutic resistance [55]. Another study demonstrated that the overexpression of p66Shc and its downstream targets, such as Eps8 and Rac1 [56], suggest an oncogenic function that may lead to the constitutive activation of a feed‐forward loop that enhances both p66Shc and NF‐κB signalling to promote tumour growth and metastasis [57].

The p66Shc can interact with various other signalling proteins, including kinases such as Akt and MAPK, scaffolding proteins like GRB2 and RAI, and adaptor molecules such as Shc and IRS, which improve the engagement and assembly of multiprotein signalling complexes and facilitate the activation of NF‐κB [58]. By connecting these signalling components, p66Shc enhances signal transduction efficiency, ensuring that the activation processes are swift and coordinated. The interaction between p66Shc and NF‐κB signalling creates a complex network that is activated in many cancers; targeting this interaction may offer a promising strategy for cancer therapy.

#### 2.1.4 Role of p66Shc in Apoptosis

The p66Shc has been identified as a critical regulator of programmed cell death, with its activity directly related to the control of oxidative stress. Upon activation, p66Shc translocates to the mitochondria, generates ROS, leads to mitochondrial dysfunction and triggers apoptotic cascades [43, 59]. Elevated ROS activate cellular signalling pathways through transcription factors such as HIF‐1α and regulate the expression of key apoptotic genes, including BNIP3, P21 and VEGF [60].

Mitochondrial dysfunction leads to the release of Cytc, key pro‐apoptotic factors, into the cytosol that bind to Apaf‐1 and ATP to form apoptosomes and facilitate caspase‐9 activation [61]. The SMAC/DIABLO in the cytosol also helps to inhibit the activity of IAPs and promote apoptosis. The activated caspase‐9 cleaves and activates caspase‐3 to execute apoptotic cell death through the nuclear lamina disassembly and DNA fragmentation [62].

p66Shc also upregulates the expression of pro‐apoptotic proteins such as BAX and BAK, which oligomerise and form pores in the outer mitochondrial membrane, facilitating the release of Cytc while inhibiting anti‐apoptotic proteins like BCL‐2 and BCL‐xL (Table 1) [30]. This shift in the balance of pro‐survival and pro‐apoptotic signals further tips the scale towards apoptosis.

These events change the associated morphological and biochemical characteristics of apoptosis, including chromatin condensation, DNA fragmentation through activated caspases, and the formation of apoptotic bodies, which are then cleared by phagocytic cells, maintaining tissue homeostasis and preventing the propagation of potentially malignant cells.

#### 2.1.5 Other Signalling Pathways and Crosstalks

The p66Shc amplifies MAPK/ERK signalling via ROS‐dependent oxidation of RAS (e.g., HRAS, KRAS), which sustains RAF/MEK/ERK activation (e.g., p‐ERK Thr202/Tyr204) [63]. ERK further phosphorylates and stabilises EMT transcription factors, such as Snail, creating a positive feedback loop that collaborates with TGF‐β signalling [64]. The Akt‐ERK contrast promoted by p66Shc can direct cells toward apoptosis (via FOXO3‐mediated BIM upregulation) or senescence, depending on the stress duration [65].

p66Shc also enhances TGF‐β‐induced EMT by stabilising SMAD4 through ROS, enabling SMAD2/3 nuclear translocation and transcriptional upregulation of EMT regulators (including Snail, Twist and ZEB1) [66]. These factors suppress epithelial markers (such as E‐cadherin) and induce mesenchymal markers (like VIM and N‐cadherin) [67]. Simultaneously, p66Shc activates the ASK1‐MKK4/7‐JNK cascade, leading to JNK phosphorylation (p‐JNK Thr183/Tyr185) and pro‐apoptotic signalling via BIM and c‐Jun [28]. JNK also phosphorylates SMAD3, enhancing TGF‐β/EMT effects, while its stabilisation of PTEN suppresses the PI3K/Akt, creating a pro‐apoptotic feedback loop [28, 29].

Metabolically, p66Shc inhibits mTORC1 via Akt suppression and AMPK‐mediated TSC2 activation, reducing anabolic processes and promoting autophagy [68, 69]. Conversely, p66Shc supports mTORC2 activity, maintaining Akt phosphorylation (Ser473) to balance survival and stress adaptation [70, 71].

In general, p66Shc activation suppresses PI3K/Akt‐mTORC1 and TCR signalling while potentiating MAPK/ERK, JNK and TGF‐β/EMT pathways, driving outcomes such as apoptosis, immune tolerance or metastatic progression. Targeting p66Shc or its downstream effectors, including ROS scavengers and JNK inhibitors, may recalibrate dysregulated signalling in cancer, fibrosis and autoimmune disorders, offering novel therapeutic avenues.

### 2.2 Interaction of p66Shc With Other Oncogenes and Tumour Suppressors

Oncogenic miR‐21 suppresses p66Shc, reducing ROS production and enhancing PI3K/Akt and MAPK/ERK signalling to promote survival and metastasis [72]. Mutually, tumour‐suppressive miR‐143 targets p66Shc, preventing ROS‐dependent activation of JNK and p53‐mediated apoptosis while inhibiting TGF‐β‐induced EMT [73]. The miR‐34a, induced by p53, forms a negative feedback loop with p66Shc, restricts ERK hyperactivation and enhances p53 pro‐apoptotic effects [74]. Meanwhile, miR‐221/222 indirectly upregulates p66Shc activity by targeting PTEN and ROS to hyperactivate PI3K/Akt/mTOR signalling and promote proliferation and therapy resistance [26, 75, 76]. Let‐7 acts opposite to these effects by suppressing RAS and ErbB receptors, indirectly attenuating growth factor‐induced p66Shc activation and downstream MAPK/ERK and PI3K/Akt pathways [26, 27].

DCLK1, a cancer stem cell marker, cooperates with p66Shc to enhance TGF‐β/Smad2/3 signalling, promoting EMT through Snail and Twist upregulation. This collaboration is reinforced by ROS‐mediated JNK activation, which supports survival under stress [77]. The tumour suppressor p53 transcriptionally upregulates p66Shc under oxidative stress, creating a pro‐apoptotic loop stabilised by ROS‐induced inhibition of MDM2 [78]. However, p53 also induces miR‐34a to suppress p66Shc, balancing ROS levels to prevent cellular damage. This interplay indirectly modulates mTOR signalling, as p53 reactivates PTEN to antagonise PI3K/Akt/mTOR, while miR‐34a‐mediated p66Shc inhibition suppresses the mTOR pathway (Table 1) [74]. PTEN is a critical target of p66Shc; ROS oxidises PTEN, inactivating its lipid phosphatase function and unleashing PI3K/Akt/mTOR signalling to promote glycolysis and angiogenesis (Table 1) [75]. The miR‐221/222‐PTEN and p66Shc enhance Akt activity and stimulate resistance to targeted therapies [79].

The retinoblastoma (Rb) protein is another node in the p66Shc network. p66Shc activates CDKs, phosphorylating Rb to release E2F transcription factors, enabling cell cycle progression (Table 1). ERK signalling, amplified by p66Shc, further phosphorylates Rb and boosts proliferation [35]. Rb inactivation also promotes EMT through E2F‐mediated upregulation of pro‐invasive factors, linking p66Shc to metastatic pathways [80]. Mortalin, a mitochondrial chaperone, sequesters p53 in the cytoplasm, but p66Shc disrupts this interaction via ROS, liberating p53 to activate pro‐apoptotic genes like BAX and NOXA [31]. Along with TGF‐β and JNK signalling, this mechanism induces apoptosis through Mortalin overexpression, which often counteracts these effects (Table 1) [31, 32].

The prolyl isomerase Pin1 enhances p66Shc activity by stabilising its phosphorylated form, promoting mitochondrial translocation and ROS production, inactivating PTEN and stimulating PI3K/Akt and ERK pathways to boost proliferation (Table 1) [33]. Pin1 also stabilises EMT transcription factors such as β‐catenin, linking p66Shc to TGF‐β and Wnt‐promoting metastasis [34]. SIRT1, a NAD^+^‐dependent deacetylase, modulates p66Shc by deacetylating it to reduce ROS generation [81]. Inversely, ROS inhibits SIRT1, activating p53 and FoxO pathways to induce senescence or apoptosis (Table 1) [36]. This mutual regulation influences ageing and stress responses through the PI3K/Akt and JNK signalling pathways.

ErbB receptors, particularly ErbB2 (HER2) and ERBB‐3, further integrate growth signals with p66Shc activity. HER2 phosphorylates p66Shc at Tyr349, promoting its mitochondrial localisation and ROS production, which amplifies the MAPK/ERK and PI3K/Akt pathways, leading to proliferation and trastuzumab resistance in HER2‐positive cancers. ERBB‐3 heterodimerises with HER2, directly binding to PI3K to activate the Akt/mTOR signalling. p66Shc, in conjunction with ERBB3 signalling, inactivates PTEN and enhances the mTOR pathway [82].

### 2.3 p66Shc Function in Different Cancer Types

The action of p66Shc in tumorigenesis is also complex and appears to change with the type of cancer, influencing tumour initiation and progression through distinct mechanisms (Table 2).

**TABLE 2:** Summary of p66Shc roles in different cancers.

Cancer type	Expression & Pattern	Main pathways involved	Functional role	Clinical implications
Breast cancer	Elevated levels correlate with aggressive phenotypes	PI3K/Akt, NF‐κB, MAPK/ERK	Promotes survival, proliferation, metastasis	Potential biomarker, therapeutic target
Colorectal	Overexpressed; associated with progression	PI3K/Akt, ERK, NF‐κB	Enhances tumour growth and invasion	Marker of poor prognosis
Lung	Variable; often decreased in late stages	Multiple pathways, context‐dependent	Early suppressor; late promoter roles	Biomarker research ongoing
Hepatocellular (HCC)	Dual roles observed; expression varies with stage	Oxidative stress, PI3K/Akt, MAPK/ERK	Both tumour‐promoting and tumour‐suppressive roles	Complex; stage‐specific targeting
Ovarian	Elevated in tumours; linked with poor prognosis	PI3K/Akt, NF‐κB, oxidative stress pathways	Promotes proliferation, resistance to apoptosis	Potential therapeutic target
Melanoma	Increased in metastatic lesions	ROS, NF‐κB, MAPK pathways	Supports invasion, metastasis	Prognostic and therapeutic relevance
Prostate	Expression varies; correlated with aggressive disease	Androgen receptor pathways, ROS, NF‐κB	Modulates growth and survival	Marker for prognosis and therapy
Pancreatic	Overexpression linked with poor survival	Multiple pathways, including ROS‐driven oxidative stress	Facilitates progression and resistance	Candidate for targeted therapy

#### 2.3.1 Breast Cancer (BC)

The role of p66Shc in BC has been well investigated due to its multifaceted involvement in tumour progression, particularly in the EMT induction [83]. Studies have demonstrated that the involvement of p66Shc in switching BC cells to a more aggressive, mesenchymal‐like phenotype involves prominent morphological changes, loss of the epithelial marker E‐cadherin and the acquisition of high capabilities for migration [84]. Overexpression of p66Shc has been identified as a prognostic biomarker associated with more aggressive disease and poorer outcomes in patients, making it an attractive target for therapeutic intervention [83, 85].

In BC cells, p66Shc drives redox signalling pathways via interaction with alpha‐1‐syntrophin (SNTA1) and Grb2 that facilitate tumour cell migration by activating the RhoA GTPase (regulator of cytoskeletal dynamics) [1, 3]. Furthermore, the mitochondrial localisation of p66Shc enables it to influence mitochondrial function and ROS production, thereby maintaining a critical balance between promoting cell survival and inducing apoptosis, which complicates the therapeutic landscape [55, 86]. Another important interaction involves p66Shc and steroid hormone signalling, especially in hormone‐sensitive BC. The upregulation of p66Shc in the presence of oestrogens has been linked to enhanced cell proliferation and the appearance of hormone‐resistant phenotypes [85, 87, 88]. The interplay would, therefore, suggest that targeting p66Shc may provide a strategic approach to managing hormone‐dependent BCs. Increased p66Shc expression has also been linked to metastatic potential in BC, particularly in lymph node‐positive tumours [85]; however, other studies showing a negative association of p66Shc expression with primary breast tumours reflect that the functional complexity of this protein is dependent on the environment and phase of the disease [85].

The interactions between p66Shc and HER2 add complexity to BC treatment in clinical implications. HER2‐positive BC patients often benefit from targeted therapies like trastuzumab; however, the presence of p66Shc may impact treatment efficacy due to its association with aggressive tumour behaviour and potential resistance mechanisms [89]. Further characterisation of p66Shc interaction with HER2 could thus pave the way toward new strategies in BC therapy for overcoming resistance and improving patient outcomes.

#### 2.3.2 Colorectal Cancer (CRC)

Among all CRC targets, p66Shc has been one of the most studied targets, particularly for its heterogeneous role in controlling oxidative stress responses and modulating EMT [90, 91]. Indeed, numerous studies have revealed that the phosphorylation of p66Shc acts as a pro‐oxidant in the mitochondrial matrix and induces the production of ROS through Cytc oxidation [92, 93], which enables tumours to grow in an oxidised environment [94]. The interaction between p66Shc and ROS is quite complicated, as elevated levels of p66Shc can increase oxidative stress, leading to the upregulation of p66Shc and creating a feedback loop that makes tumours more aggressive [56, 95].

EMT is a critical process in tumour metastasis characterised by the loss of epithelial characteristics and the acquisition of mesenchymal traits, which enhance tumour cells' migratory and invasive capabilities [96]. Several studies have shown that p66Shc regulates the levels of EMT transcription factors, including Snail and Slug, which promote the invasive phenotype of CRC cells [97, 98]. The interaction of p66Shc with oncogenic factors, such as Aurora‐A, enhances Slug activity, suggesting crosstalk with critical oncogenic pathways that strengthens its potential as a therapeutic target in CRC [97, 99]. However, p66Shc is often associated with tumour progression; its absence can also enhance tumorigenic potential through ROS‐dependent mechanisms, revealing a more complex role in CRC biology [53].

The p66Shc also suppresses antioxidant genes (such as SOD1, GPX, etc.), upregulates heat shock proteins (including HSP70 and HSP90) and enhances cellular stress balance to promote a pro‐oxidative tumour environment [100]. This oxidative imbalance is exacerbated by the downregulation of p53 expression through p66Shc, which disrupts tumour‐suppressive functions, intensifies oxidative damage and accelerates tumour progression [101]. The p66Shc also influences mesenchymal stem cells within the tumour microenvironment (TME), promoting their proliferation and inducing genetic alterations that enhance cancer stemness and metastatic behaviour [102]. Targeting p66Shc presents a dual opportunity to reduce oncogenesis and feedback p53‐mediated tumour suppression while concurrently interrupting tumour–stromal interactions. Inhibition of redox regulation, p53 signalling and stem cell dynamics can inform novel strategies to improve therapeutic efficacy and overcome chemoresistance in CRC.

#### 2.3.3 Lung Cancer

The expression of p66Shc in lung cancer, particularly in non‐small cell lung carcinoma (NSCLC), is typically linked to advanced stages of the disease. Mechanistic studies have shown that p66Shc promotes lung tumourigenesis by modulating the PI3K/Akt and MAPK pathways, which enhance cell proliferation and survival in the hypoxic conditions characteristic of the TME [20, 103, 104]. This protein can impact the effectiveness of targeted therapies due to its role in regulating apoptotic processes, making it a significant factor in therapy resistance.

Research also indicates that p66Shc facilitates lung tumorigenesis through heightened activation of the PI3K/Akt pathway [20, 103, 104]. The interplay between these pathways is crucial, as they often influence downstream effectors and modulate oncogenic processes. Continuous stimulation of these signalling cascades leads to the progression of NSCLC and resistance to therapies [105, 106]. Studies have demonstrated that overexpression of p66Shc correlates with enhanced activation of the PI3K/Akt pathway, which is frequently associated with resistance to targeted therapies, such as tyrosine kinase inhibitors (TKIs) [105, 106]. This condition is highly hypoxic, the hallmark of many solid tumours, including NSCLC, which may further promote tumorigenesis. Transcription of p66Shc is induced mainly under hypoxic conditions with enhanced production of ROS [107, 108] that contribute to tumour growth and complicate the treatment approach since many forms of stimulated survival signalling have turned cancer cells resistant to conventional therapies [109]. The p66Shc can also promote EMT and enhance the cell's migratory and invasive abilities in lung cancer by activating the PI3K/Akt and MAPK pathways [20, 104]. This transition is often linked to a more aggressive tumour phenotype and is associated with poorer clinical outcomes in patients with NSCLC [103]. So, targeting the PI3K/Akt and MAPK pathways, and p66Shc inhibition, may effectively overcome resistance mechanisms and improve patient outcomes [105, 106, 109].

#### 2.3.4 Melanoma

As a redox protein, the p66Shc expression differs among subtypes of melanomas, which may correlate with tumour invasiveness and/or genetic backgrounds, including BRAF mutation status [110]. Approximately 50% of melanomas harbour activating mutations of BRAF, with the V600E variant being the most common [110]. This variant is associated with tumour‐aggressive phenotypes and possibly impacts p66Shc expression [110, 111]. For example, in melanomas bearing mutated BRAF, p66Shc may increase the sensitivity of the cells to oxidative stress‐induced apoptosis and may function as a tumour suppressor [112, 113]. In contrast, the upregulation of this protein increases cell motility and invasiveness, revealing that its role in melanoma subtypes is complicated [112, 114]. Thus, combinatory approaches with BRAF/MEK inhibitors or ROS‐inducing agents (e.g., chemotherapy) are potential avenues, though the dependent roles of p66Shc necessitate careful evaluation.

p66Shc also enhances melanoma invasiveness by regulating cytoskeletal dynamics and matrix metalloproteinase (MMP) activity. ROS generated by p66Shc activate MMPs, facilitating extracellular matrix degradation [115]. Furthermore, p66Shc may promote EMT‐like transitions through Rho GTPase signalling, augmenting migratory capacity and metastatic invasion [116]. The interplay of p66Shc with other pathways, for instance, arginase II, further complicates the issue. Arginase II promotes melanoma cell migration and adhesion by mechanisms that involve p66Shc and increased production of hydrogen peroxide [114], suggesting that p66Shc controls melanoma invasiveness and oxidative stress responses. Also, since p66Shc interacts with tumour suppressor proteins such as p53, evidence confirms its involvement in broader apoptotic signalling pathways, particularly those responding to oxidative stress [113]. Such findings strengthen the complexity of p66Shc's roles, further emphasising the need for research that explains, in detail, how such proteins function and are regulated in melanoma.

#### 2.3.5 Prostate Cancer (PCa)

The p66Shc expression in PCa is often upregulated in response to androgens, the primary hormones driving PCa growth, through androgen receptor (AR) signalling pathways, and creates a microenvironment conducive to tumour growth [117, 118]. Steroid hormones, such as dihydrotestosterone (DHT), upregulate p66Shc protein levels and increase PCa cell proliferation, which can be inhibited by androgen antagonists such as Casodex [119]. Additionally, p66Shc is negatively regulated by miRNAs, such as miR‐29b, while its downregulation in PCa tissues correlates with increased p66Shc levels and enhances cancer cell survival and proliferation [117, 118].

The p66Shc interaction with IRS proteins and the PI3K/Akt pathway enhances signalling cascades in cancer progression. It also activates the ERK pathway and increases cell migration and invasion [120, 121]. As a redox sensor, p66Shc activation induces DNA damage and promotes tumorigenesis through ROS production while activating survival pathways that help cancer cells adapt to a hostile microenvironment [4, 17, 82]. Furthermore, the role of p66Shc in apoptosis is critical for determining the response of PCa cells to therapies, including androgen deprivation therapy (ADT). It can influence therapeutic sensitivity/resistance by modulating apoptotic pathways and highlights its therapeutic target potential in PCa [117, 118].

#### 2.3.6 Hepatocellular Carcinoma (HCC)

The role of p66Shc has been linked to various clinical outcomes in HCC, suggesting its potential as both a prognostic biomarker and a therapeutic target [122, 123]. Recent studies indicate that low p66Shc levels, in conjunction with high SerpinB3 levels, promote necroptosis [87], which has been correlated with better survival outcomes in HCC patients, suggesting that the balance between these two proteins is crucial for determining cell fate in the TME [87].

Furthermore, p66Shc elevated expression has been observed in liver injury models, including those related to alcohol and non‐alcoholic fatty liver disease (NAFLD), indicating its role in liver fibrosis and carcinogenesis [124]. Its interaction with mitochondrial dynamics through translocation also significantly influences apoptotic pathways [125]. Additionally, p66Shc is implicated in metabolic pathways, particularly in insulin signalling and hepatic steatosis, which are pioneers of HCC [126]. Environmental factors, including dietary components, can modulate p66Shc expression, suggesting potential therapeutic avenues [127]. Additionally, targeting p66Shc through siRNAs has shown promise in reducing oxidative damage and improving liver function in preclinical models [125].

The involvement of p66Shc in signalling pathways, such as STAT3, has highlighted its role in HCC. The knockdown of p66Shc has been shown to inhibit HCC progression by modulating the TME through the STAT3 signalling pathway [128]. So, p66Shc not only contributes to oxidative stress but also acts as a signalling hub that can influence the behaviour of HCC cells. The interplay between p66Shc and other signalling molecules, including those involved in autophagy and cell survival, such as mTOR, AMPK, BCL‐2 family proteins and the PI3K/Akt pathway, complicates its role in HCC biology [129].

#### 2.3.7 Pancreatic Cancer (PC)

In PC, p66Shc expression is often upregulated, correlating with increased oxidative stress and enhanced tumour aggressiveness. For example, studies have shown that p66Shc influences apoptotic pathways within pancreatic cells by promoting ROS levels [130, 131]. In addition to p66Shc phosphorylation [131], which promotes proliferation and metastatic potential [130], the interplay with other downstream signalling pathways further elucidates its role in PC [132]. For example, the interaction between p66Shc and secreted TGF‐β1 from pancreatic stellate cells plays a significant role in enhancing the stemness and tumorigenicity of PC cells [130], which highlights the mediating role of p66Shc in the TME and facilitating crosstalk between cancer cells and stromal components.

Recent studies suggest that modulating p66Shc activity through strategies such as small‐molecule inhibitors or RNAi could lower ROS levels, thereby increasing the sensitivity of PC cells to chemotherapy [46]. This highlights the possibility of p66Shc not only as a target for treatment but also as a biomarker for early detection and prognosis in PC patients. Moreover, the relationship between p66Shc and metabolic pathways in PC is under investigation. The upregulation of p66Shc in response to metabolic stressors, including high‐fat diets and hypoxia, indicates its role in the metabolic reprogramming of cancer cells [133]. This metabolic shift is characterised by enhanced glycolytic activity and altered mitochondrial function, which are recognised features of cancer cell metabolism.

#### 2.3.8 Ovarian Cancer (OC)

One of the critical functions of p66Shc is its role in mediating oxidative stress, which is a significant factor in the pathophysiology of OC. For instance, Wang et al. highlighted that suppression of p66Shc can prevent hyperandrogenism‐induced oxidative stress in ovarian tissues associated with OC [134]. The study indicated that the expression levels of p66Shc are elevated in OC, which correlates with increased ROS production and enhanced tumour aggressiveness [134].

The activation of MAPK signalling pathways, particularly the dysregulated PI3K/Akt pathway, is also relevant in OC, with p66Shc implicated in modulating these pathways [135, 136]. This interaction positions p66Shc as a potential biomarker for OC progression and a target for therapeutic strategies aimed at disrupting these signalling networks. Onnis et al. reported that p66Shc acts as a negative regulator of cell survival, which could be particularly beneficial in overcoming the resistance to often‐seen apoptosis in ovarian tumours [129]. Additionally, the p66Shc relationship with integrins is crucial for cell adhesion and migration [137], suggesting that targeting p66Shc could be a strategic approach to preventing metastasis in OC. Inhibiting p66Shc activity or downregulating its expression may reduce oxidative stress and improve the efficacy of chemotherapeutic agents [4].

## 3 The p66Shc Dual Role: Tumour Suppressor Versus Oncogene

**FIGURE 3:**
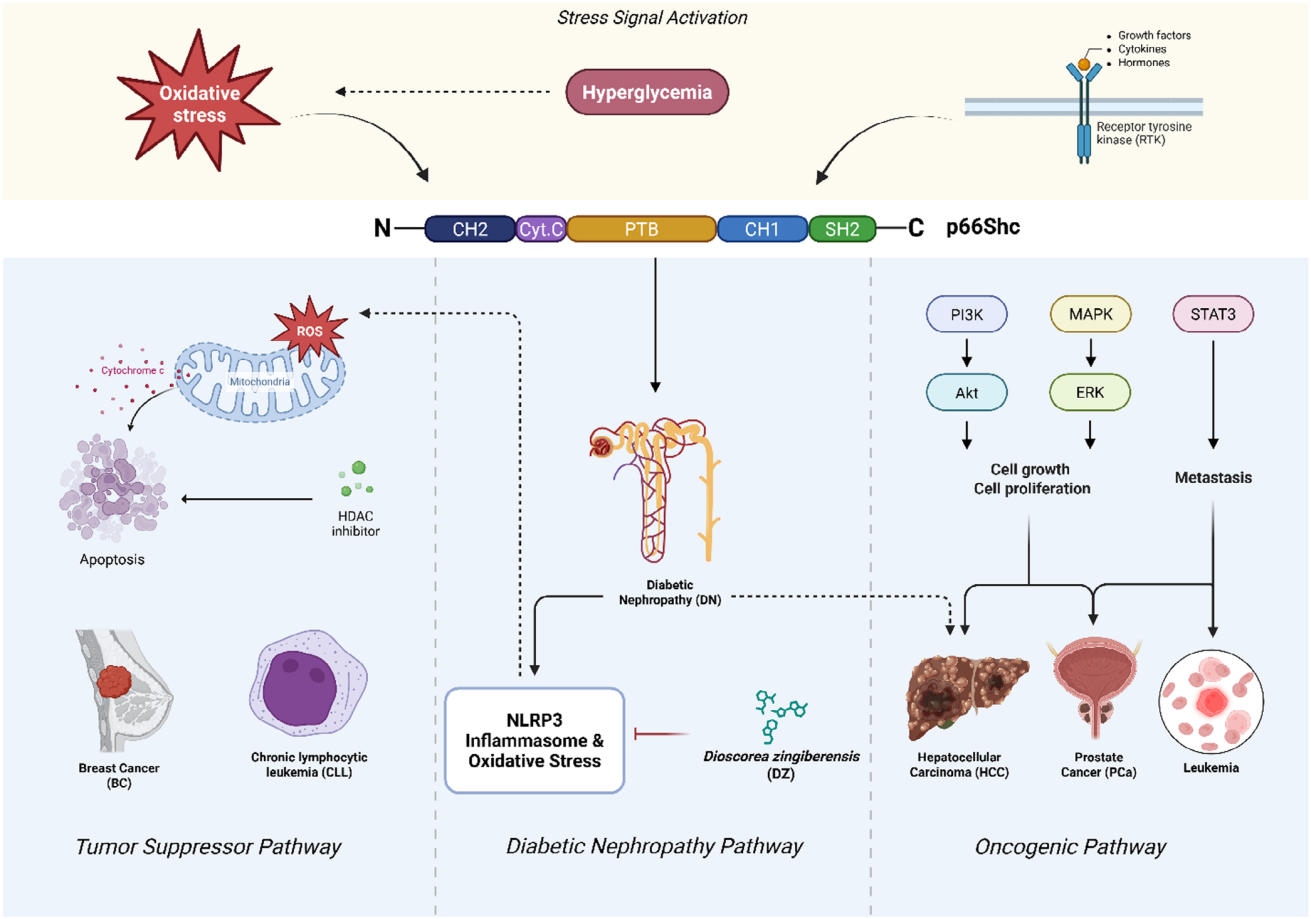
p66Shc's roles in cancer and related diseases: Adaptive or variable functions. This schematic illustrates the structural domains of p66Shc and their activation by various stress signals, including ROS and RTKs. It depicts the dual roles of p66Shc in cellular physiology: (1) as a tumour suppressor—mediating mitochondrial ROS production and Cytc release to promote apoptosis in BC and CLL, with this function being potentiated by HDAC inhibitors; and (2) as an oncoprotein—activating proliferative and metastatic pathways via PI3K/Akt, MAPK/ERK and STAT3 signalling cascades in HCC, PCa and leukaemia. The figure further highlights the impact of pharmacological modulation, such as HDAC inhibitors in CLL and *Dioscorea zingiberensis* (DZ) in diabetic nephropathy (DN), where p66Shc inhibition attenuates NLRP3 inflammasome activity and oxidative stress, potentially influencing cancer‐related comorbidities, including HCC. Created with BioRender.com [138, 139, 140].

The p66Shc plays a paradoxical dual role as a tumour suppressor and oncogene in cancer research (Figure 3).

### 3.1 Mechanisms and Circumstances Influencing Tumour‐Promoting and Tumour‐Suppressive Functions

The p66Shc plays a central role in enhancing mitochondrial ROS, which triggers Cytc release and caspase‐dependent apoptosis and effectively purges cells with irreversible DNA damage. This pro‐apoptotic function is synergistically enhanced by interaction with p53 [101]. p66Shc stabilises p53 by blocking MDM2‐mediated ubiquitination, thereby upregulating the transcription of pro‐apoptotic genes, like BAX and PUMA [101]. Concurrently, p66Shc induces senescence by upregulating the cell cycle inhibitor, p21, which arrests the proliferation of pre‐cancerous cells [138]. Beyond apoptosis and senescence, p66Shc suppresses oncogenic signals by interacting with growth factor receptors such as EGFR and restricts hyperactive RAS/MAPK and PI3K/Akt pathways that promote proliferation [1]. These mechanisms position p66Shc as a guardian in early tumorigenesis, counteracting genomic instability and mitogenic hyperactivity to maintain cellular integrity.

In progressive malignancies, p66Shc paradoxically adopts oncogenic properties that enhance tumour survival and progression. Under chronic oxidative stress, p66Shc shifts from inducing apoptosis to promoting adaptive survival by activating PKCβ, which phosphorylates and inactivates pro‐apoptotic proteins, like BAD [1]. This survival advantage is then completed by its role in metabolic reprogramming. p66Shc stabilises HIF‐1α to promote the Warburg effect and enhance glycolytic flux even under normoxic conditions to supply biosynthetic intermediates for rapid tumour growth [141, 142]. Additionally, p66Shc stimulates immune evasion by upregulating PD‐L1 through NF‐κB signalling and enabling cancer cells to escape T‐cell destruction [52]. It also collaborates with stromal components to secrete pro‐angiogenic factors such as VEGF to facilitate the formation of nutrient‐rich vascular networks that sustain tumour expansion [143]. These adaptations highlight p66Shc's capacity to utilise stress pathways in established tumours, transitioning from a suppressor to a facilitator of malignancy by enhancing survival, metabolic fitness and microenvironmental crosstalk.

### 3.2 Adaptive or Variable Functions of p66Shc

The dual role of p66Shc as both a tumour suppressor and an oncogene is highly condition‐dependent, with discrepancies in its pro‐ versus anti‐apoptotic effects observed across various tumour types and microenvironments. In BC, high p66Shc expression enhances chemotherapy sensitivity by promoting ROS‐mediated apoptosis, particularly in triple‐negative breast cancer (TNBC) [144]. Conversely, in hormone‐sensitive BC, p66Shc upregulation correlates with oestrogen‐driven proliferation and therapy resistance, suggesting an oncogenic role [87]. In CRC, the pro‐oxidant activity of p66Shc drives EMT and metastasis through the upregulation of Snail and Slug, yet its absence can paradoxically enhance tumorigenesis via ROS‐dependent NF‐κB activation [53]. These conflicting roles may stem from differences in ROS levels, with low‐to‐moderate ROS promoting survival signalling via the PI3K/Akt pathway, while high ROS triggers apoptosis. Tumour stage also influences p66Shc function; early‐stage tumours may benefit from its tumour‐suppressive effects, whereas advanced tumours exploit its survival and metastatic potential. Microenvironmental factors, such as hypoxia in NSCLC, further modulate p66Shc activity by enhancing ROS production and PI3K/Akt signalling [20]. These discrepancies highlight the need for further research to elucidate the molecular factors, such as signalling pathway dominance, post‐translational modifications and TME interactions, that govern p66Shc's role in specific cancers.

## 4 p66Shc as a Biomarker and Therapeutic Target

### 4.1 Prognostic Significance of p66Shc Expression in Cancers

The differential expression of p66Shc across various tumour types has accumulated significant attention, particularly regarding its potential as a therapeutic target and prognostic biomarker [1]. Elevated levels of p66Shc are associated with aggressive tumour characteristics, including increased proliferation, migration and resistance to apoptosis in several malignancies, such as prostate, breast, colorectal and lung cancers [145]. Indeed, in PCa, high p66Shc expression identifies tumours with high metastatic potential; thus, targeting this protein impacts disease outcome. Indeed, in BCs, especially those belonging to the HER2‐positive subtype, a high level of p66Shc expression is associated with a poorer clinical outcome, suggesting that it may hold potential for use in risk stratification and personalised treatment strategy [3]. These results outline the role of p66Shc as a potential biomarker for risk assessment and personalised therapy. The role of p66Shc in the advanced stage of disease and poor prognosis of CRC has also been described, which indicates that p66Shc can modulate the typical oxidative stress and apoptosis in cancer [88]. In addition, the association of Shc proteins with the PI3K/Akt intracellular signalling cascade underlines their potential role as prognostic biomarkers [146]. In CRC cells, p66Shc expression is related to tumour aggressiveness; therefore, the Shc adaptor can provide information on disease prognosis [147]. In lung cancer, studies have demonstrated the role of p66Shc in inducing anchorage independence in NSCLC, an important feature in defining metastatic behaviour [148]. The increased expression of p66Shc in NSCLC is associated with the induction of oxidative phosphorylation that significantly improves the energy supply and survival of neoplastic cells under metabolic stress [149]. This relationship offers p66Shc as an interesting candidate biomarker for prognosis evaluation and choosing the optimal therapy [148].

Beyond individual cancer types, p66Shc expression has implications in other malignancies, such as OC, associated with ROS‐generating enzymes that influence tumour behaviour [85, 88]. The mechanistic role of p66Shc in cancer progression is complex, primarily because it serves as a mediator of oxidative stress responses via mitochondrial pathways [150], leading to increased ROS; this influences signalling cascades related to chronic inflammation and metabolic dysregulation, which promote tumour growth [151]. Additionally, p66Shc involvement in regulating apoptosis indicates its potential as a prognostic biomarker [152]. Low expression of p66Shc in chronic lymphocytic leukaemia (CLL) was associated with poor prognostic outcomes, reflecting its role in B‐cell survival and tumour development [153], and suggests that evaluating p66Shc levels could provide valuable prognostic information in CLL and potentially other haematological malignancies.

### 4.2 Current Therapeutic Strategies Targeting p66Shc

The dual role of p66Shc in cancer is influenced by therapeutic strategies. In early‐stage tumours, p66Shc approaches focus on enhancing its pro‐apoptotic activity. For example, gene therapy to restore p66Shc expression in cancers (e.g., CRC) with its downregulation has shown preclinical promise by reactivating apoptosis and senescence [154]. Conversely, in advanced malignancies, p66Shc adopts oncogenic roles (RNA interference (siRNA/shRNA) or CRISPR‐based silencing) to limit its survival‐promoting effects, as seen in TNBC [144]. Therapeutic synergy is also achieved by targeting p66Shc interaction partners; in p53‐wildtype cancers, MDM2 antagonists like Nutlin‐3 increase p53 to induce apoptosis [155], while kinase inhibitors such as EGFR or Akt inhibitors prevent resistance by leveraging p66Shc suppression in the RAS/MAPK and PI3K pathways. Post‐translational modifications (PTMs) of p66Shc offer other treatments, such as inhibiting PKCβ‐mediated phosphorylation at Ser36 with compounds like enzastaurin, which blocks its oncogenic signalling, whereas modulators of acetylation (e.g., SIRT1 activators or inhibitors) adjust its mitochondrial localisation [156]. ROS‐based therapies also highlight the duality of targeting p66Shc; antioxidants like piperlongumine enhance apoptosis in early‐stage tumours [157], while N‐acetylcysteine may mitigate chronic oxidative stress [158] that promotes pro‐survival activity in advanced disease. The p66Shc modulation, in combination with immunotherapy, includes strategies such as anti‐PD‐1 to prevent PD‐L1 upregulation [159] or anti‐angiogenic agents, such as bevacizumab, to disrupt stromal support and immune evasion [160]. However, these approaches require accurate biomarker personalisation, including p53 status, ROS levels and PD‐L1 expression, to avoid paradoxical effects. Challenges remain in ensuring tumour‐specific delivery, avoiding off‐target consequences of p66Shc activation in metastatic conditions, and advancing preclinical candidates to clinical trials. Despite these hurdles, the dynamic role of p66Shc positions it as a hub therapeutic node.

### 4.3 Potential for Novel Targeted Therapies

Targeting p66Shc opens up new, exciting perspectives in designing innovative therapeutic strategies for treating cancer (Table 3).

**TABLE 3:** Current and emerging therapeutic strategies targeting p66Shc.

Therapeutic strategy	Type	Mechanism of action	Stage of development	Clinical trial status
Small molecule inhibitors	Small molecule	Inhibits p66Shc activity	Preclinical	Not yet initiated
CRISPR/Cas9 gene editing	Gene editing	Knock‐out of the p66Shc gene	Early phase	Ongoing trials
Combination with immunotherapy	Combination therapy	Enhances immune response against tumours	Clinical phase	Active trials
Antisense oligonucleotides	Nucleic acid therapy	Reduces p66Shc expression	Preclinical	Not yet initiated

#### 4.3.1 Small Molecule Inhibitors

Small molecule inhibitors targeting p66Shc aim to disrupt its pro‐tumorigenic functions by blocking phosphorylation‐dependent activation, interfering with protein interactions or modulating redox signalling [6]. For instance, p66Shc mitochondrial translocation, ROS generation and inhibitors like ruboxistaurin, a PKCβ inhibitor approved for diabetic retinopathy, have shown promise in preclinical studies, suppressing lung tumour growth when combined with cisplatin [161]. Similarly, resveratrol, a natural polyphenol, indirectly downregulates p66Shc transcription through SIRT1 activation, thereby reducing metastasis and chemoresistance in BC models [162]. Mitochondria‐targeted antioxidants like MitoTEMPO, which balance ROS, have also demonstrated anti‐tumour effects in melanoma by oxidative stress inhibition [163]. Additionally, synthetic peptidomimetics can bind to the p66Shc CH2 domain interactions with Cytc, which have induced apoptosis in CRC cells and sensitised them to chemotherapy [164].

In CLL, the loss of p66Shc paradoxically drives leukemogenesis by altering chemokine receptor profiles, suggesting that interventions to restore apoptotic signalling in pro‐survival niches may be beneficial [153, 165]. Preclinical studies have highlighted the synergistic anti‐tumour effects of combining p66Shc inhibitors with anti‐IL‐9 monoclonal antibodies, disrupting tumour‐supportive microenvironments [165, 166]. Emerging strategies include USP15 inhibitors (targeting p66Shc stability) [122] and simvastatin analogues that block p66Shc‐Akt/ERK interactions [8, 167], offering novel therapeutic entry points. In PCa, p66Shc facilitates the transition to castration‐resistant states [167, 168], underscoring its relevance in advanced disease; however, deeper mechanistic insights into its role in metastasis are needed.

Despite these preclinical advances, no p66Shc‐specific inhibitors have entered clinical trials for cancer due to several challenges, including achieving selectivity over the physiologically essential p52/p46Shc isoforms, balancing ROS suppression to avoid compromising ROS‐dependent chemotherapies, and validating p66Shc as a predictive biomarker. Repurposed agents like bardoxolone methyl (an Nrf2 activator in Phase III trials for kidney disease) [169] and dasatinib (a kinase inhibitor with off‐target effects on p66Shc phosphorylation) [170] offer near‐term opportunities to explore p66Shc modulation in oncology. Combining p66Shc inhibitors with chemotherapy may enhance efficacy by attacking multiple survival pathways [8, 168], while multilevel approaches could address complex signalling in resistant tumours, such as targeting miR‐34a‐5p (which regulates p66Shc and apoptosis) [171]. Future efforts should prioritise high‐throughput screening for isoform‐selective inhibitors, nanoparticle‐based delivery to enhance mitochondrial targeting, and early‐phase trials testing repurposed agents in cancers with high p66Shc expression, such as TNBC. To date, no registered clinical trials have directly targeted p66Shc, which underscores the need for collaborative translational research to bridge the preclinical promise into therapeutic reality.

#### 4.3.2 Gene Editing Approaches (CRISPR/Cas9 Technology)

CRISPR/Cas9 technology enables the precise editing of the p66Shc gene, which alters its expression and function. For instance, studies have shown that p66Shc is regulated by various pathways, including the SIRT1/p66Shc axis, which is crucial in mediating oxidative stress responses and apoptosis in endothelial cells [74, 172]. By employing CRISPR/Cas9 to knock out or modify the p66Shc gene, researchers can investigate its role in these pathways and potentially mitigate the adverse effects associated with its overexpression, such as increased ROS production and apoptosis [1, 173]. Moreover, p66Shc's involvement in cellular signalling pathways makes it a strategic target for gene editing. For example, the expression of p66Shc is influenced by epigenetic modifications, such as histone acetylation and methylation, which can be manipulated through CRISPR/Cas9 to enhance or inhibit its expression [174]. This approach could provide insights into the mechanisms by which p66Shc contributes to diseases like diabetes and cancer, where its dysregulation is often observed [123, 175].

In chronic diseases, targeting p66Shc with CRISPR/Cas9 could offer therapeutic benefits. For instance, in models of diabetic endothelial dysfunction, the deletion of p66Shc has been shown to alleviate oxidative stress and improve vascular function [74, 172]. Similarly, manipulating p66Shc expression in cancer research could alter tumour cell proliferation and survival, as evidenced by studies demonstrating that high levels of p66Shc correlate with increased oxidative damage and reduced cell viability in cancer cells [176, 177]. Furthermore, the potential for CRISPR/Cas9 to create animal models with specific p66Shc modifications can facilitate the study of its role in various biological processes and diseases. For example, p66Shc knockout mice have been used to demonstrate the gene's involvement in ageing and oxidative stress responses, providing a framework for understanding its function in vivo [132, 178]. Such models are invaluable for testing new therapeutic strategies for diseases where p66Shc is implicated.

#### 4.3.3 Antisense Oligonucleotides (ASOs)

ASOs [179], synthetic nucleic acid sequences designed to silence gene expression, selectively suppress p66Shc by hybridising with its SHC1 mRNA transcript [180]. They leverage mechanisms such as RNase H‐mediated degradation, translational inhibition or splice modulation to block the production of the oncogenic p66 isoform while preserving physiological p52/p46Shc variants [7, 179]. For example, gapmer ASOs engage RNase H to cleave the p66Shc transcript, steric‐blocking ASOs prevent ribosomal assembly and splice‐switching ASOs exclude exon 2 to favour shorter Shc isoforms [181]. Splice‐switching ASOs, in particular, alter mRNA processing to suppress the oncogenic isoform, a strategy validated in recent studies [182]. Beyond p66Shc, ASOs targeting oncogenes like BIRC5 and Hsp27 have demonstrated preclinical efficacy [183, 184]. BIRC5‐directed ASOs reduced mRNA levels in liver cancer cells [184], while OGX‐427, an ASO targeting Hsp27, induced apoptosis in PC models [183]. In BC cells, p66Shc interacts with alpha‐1‐syntrophin, forming a complex that activates the RhoA GTPase; targeting this interaction with ASOs could disrupt redox signalling and inhibit metastasis, as demonstrated in preclinical studies [3]. Similarly, in hepatocellular carcinoma, inhibition of p66Shc‐mediated oxidative stress via ASOs has shown the potential to alleviate fibrotic progression and improve responses to existing therapies [123]. Additionally, miR‐21‐targeting ASOs indirectly downregulate p66Shc by suppressing ROS, reducing tumour growth and liver metastasis [72].

Overcoming drug resistance, as seen with EGFR‐targeted ASOs that enhance efficacy in erlotinib‐resistant non‐small cell lung cancer [185]. However, clinical translation faces challenges like poor bioavailability [186]. Advances in delivery systems, such as conjugating ASOs to phototherapeutic nanoparticles, enhance tumour‐specific delivery while enabling synergistic photothermal therapy [187]. Chemical modifications to ASO backbones further enhance stability and biodistribution [188], building on FDA‐approved ASO therapies for genetic disorders [189]. Combination strategies such as pairing p66Shc‐targeted ASOs combined with PD‐L1 inhibitors have achieved robust responses in head and neck squamous cell carcinoma, illustrating the power of dual‐pathway targeting [188, 190]. Such approaches enhance antitumour effects and reshape the TME by modulating angiogenesis and immune evasion [191].

#### 4.3.4 Combination Therapies With Immunotherapy

Integrating p66Shc‐targeted therapies with immunotherapy offers a strategic approach to prevent immunosuppression and enhance antitumour immunity. p66Shc promotes a TME enriched with regulatory T‐cells and PD‐L1 expression, suppressing T‐cell activity [159]. Inhibitors of the PI3K/Akt pathway, such as idelalisib (a PI3Kδ inhibitor), alpelisib (a PI3Kα inhibitor) and Akt inhibitors like MK‐2206 and AZD5363, disrupt oncogenic signalling while indirectly modulating p66Shc activity [192, 193]. For instance, Akt inhibitors suppress p66Shc phosphorylation, thereby reducing ROS production and PD‐L1 upregulation and sensitising tumours to immune checkpoint inhibitors (ICIs) like anti‐PD‐1/PD‐L1 [194]. Preclinical studies demonstrate this synergy in TNBC models; alpelisib combined with anti‐PD‐1 antibodies reduced tumour growth by downregulating p66Shc‐dependent ROS and enhancing CD8^+^ T‐cell infiltration [195], while AZD5363, combined with anti‐PD‐L1, delayed castration‐resistant PCa progression in vivo by mitigating ROS‐mediated immunosuppression [196]. Early‐phase clinical trials, such as copanlisib (a PI3Kα/δ inhibitor) combined with nivolumab (NCT03795610) in solid tumours [197] and alpelisib with pembrolizumab (NCT03772561) in TNBC, highlight the translational potential of these combinations; however, efficacy remains variable without biomarker stratification. Current challenges include overlapping toxicities (e.g., hepatotoxicity), compensatory pathway activation and the lack of validated biomarkers linking p66Shc/ROS levels to therapeutic response. Future directions emphasise biomarker‐driven trials using p66Shc expression to select patients for therapies like AZD5363 [198] with durvalumab [199], novel combinations such as idelalisib with CAR‐T cells in B‐cell malignancies [200], and nanoparticle co‐delivery systems to enhance tumour‐specific targeting of agents like GSK2141795 (PI3K/mTOR inhibitor) (Table 4) [201]. By elucidating p66Shc's role in regulating immunogenic pathways such as STING/IFN‐γ [200], researchers aim to refine these strategies, ultimately bridging the gap between preclinical promise and clinical success in ROS‐high malignancies [149, 153]. Research on p66Shc in immunotherapy raises critical issues about the timing and sequencing of treatments, thereby highlighting the need for preclinical studies to identify the optimal combination modalities.

**TABLE 4:** Potential inhibitors of p66Shc and the related mechanism.

Category	Inhibitor	Mechanism	Influence on p66Shc	References
PI3K inhibitors	Idelalisib (Zydelig)	Inhibits PI3K delta.	Likely alters p66Shc‐mediated oxidative stress signalling, affecting apoptosis.	[200]
	Alpelisib (Piqray)	Inhibits PI3K alpha.	Potentially upregulates p66Shc activity in HR‐positive breast cancer.	[193]
	Copanlisib (Aliqopa)	Inhibits multiple PI3K isoforms.	Alters p66Shc signalling, enhancing apoptotic responses in lymphomas.	[197]
Akt inhibitors	MK‐2206	Allosterically inhibits Akt.	Potentially enhances apoptosis via p66Shc signalling.	[192]
	AZD5363	Inhibits Akt.	Potentially increases p66Shc activity, promoting apoptosis.	[198]
	GSK2141795	Inhibits Akt.	Disrupts p66Shc pathways, affecting cell survival and apoptosis.	[201]
	Ipatasertib	Selectively inhibits Akt.	May modulate p66Shc activity, impacting therapeutic resistance.	[202]
Broad Akt pathway modulators	Perifosine	Inhibits Akt.	Potentially influences p66Shc signalling and enhances efficacy by modulation of oxidative stress.	[203]

### 4.4 Therapeutic Implications

Efforts to therapeutically target p66Shc have gained momentum, with preclinical and clinical studies exploring its potential as a therapeutic target and biomarker. Small‐molecule inhibitors targeting p66Shc's SH2 or PTB domains have shown promise in disrupting its interactions with RTKs, inhibiting PI3K/Akt and MAPK/ERK signalling in NSCLC and BC models [20]. Additionally, inhibitors of p66Shc phosphorylation have demonstrated efficacy in reducing ROS production and tumour aggressiveness in CRC models [93]. ASOs targeting p66Shc mRNA have been shown to reduce tumour growth and enhance chemotherapy sensitivity in OC preclinical models [134]. Nanoparticle‐encapsulated siRNAs targeting p66Shc have also demonstrated efficacy in HCC, reducing oxidative damage and improving therapeutic outcomes [125].

Preclinical studies have identified p66Shc as a potential biomarker for chemotherapy response in various cancers, including BC and CRC [83]. The underlying hypothesis is that p66Shc levels could predict a tumour's sensitivity or resistance to a specific chemotherapeutic agent by modulating cellular oxidative stress. However, this concept is still in the research phase and has not yet been validated or implemented for patient stratification in large‐scale clinical trials [83]. Combining p66Shc inhibitors with ROS‐inducing agents, such as doxorubicin, or targeted therapies, like trastuzumab in HER2‐positive BC, has shown synergistic effects in overcoming resistance [89]. Similarly, combining p66Shc inhibitors with ROS‐inducing agents enhances apoptosis in melanoma with BRAF mutations, suggesting broader applicability across cancer types [112].

Recent research suggests that p66Shc plays a significant role in modulating the TME, potentially improving the effectiveness of immunotherapies [159], which underscores the need for further investigation into combination therapies, such as itraconazole, that act as a therapeutic agent targeting the TME, and indomethacin, which targets p66Shc, particularly in CRC cell lines [204]. These advances highlight p66Shc's potential as a versatile therapeutic target, warranting further clinical investigation.

## 5 Challenges and Future Directions

### 5.1 Limitations of Current Studies on p66Shc in Cancer

Several limiting factors hamper current research on p66Shc, which prevents a complete understanding of its role in cancer. A primary concern is the dependence on heterogeneous models of cancer, which yield conflicting findings regarding the role played by p66Shc across different tumour types. For instance, there is marked variation in the level of p66Shc expression among different BC cell lines, high in MDA‐MB‐231 and low in MCF‐7 cells [3]; this makes generalisations about the role that p66Shc might play in tumour progression and therapeutic resistance challenging [1]. The timing of p66Shc expression is also not defined, which raises discussion about its role in cancer. Some evidence suggests that p66Shc is a pro‐tumorigenic factor in aggressive tumours, while others have suggested it may function as a tumour suppressor [88]. In PCa, p66Shc induces apoptosis through ROS production, whereas in specific cancer cells, this protein has been associated with enhanced proliferation [205, 206]. This dichotomy highlights the importance of considering various cellular and environmental conditions that influence the function of p66Shc.

Another major drawback is the lack of longitudinal analyses in most studies; the level of p66Shc is measured only once, which is a cross‐sectional approach that does not consider dynamic changes during cancer progression and therapy [86]. Methodological improvements lead to complications in research findings. The various methods used for measuring the expression of p66Shc include immunohistochemistry, Western blotting and RT‐PCR, which may yield different results that are hardly comparable with each other [96]. Standardised protocols will allow a more reliable assessment of the level and activity of p66Shc in cancer research. Second, focusing on selected tumour types excludes the evaluation of p66Shc from a broader analysis that has been gained from other pathologies. Though most studies are intensive in BC and PCa, very few studies relate to other tumours like colorectal and gastric cancers [207]. Further research encompassing a wide array of neoplastic conditions may provide comprehensive insights into how p66Shc modulates tumour biology.

### 5.2 Need for Standardised Methodologies in p66Shc Research

Another major problem in the investigation of p66Shc in cancer is the lack of uniform methodologies. Variability in selecting cancer cell lines and methods of manipulating p66Shc expression, including overexpression versus knockdown and different assay techniques, generates inconsistent and often contradictory results that hinder the understanding of p66Shc's role in oncogenesis [1, 88]. For example, although p66Shc regulates the proliferation of many cancer cell lines, the outcomes differ dramatically depending on the cell lines and experimental conditions [1, 88]. This variability necessitates the development of standardised protocols for measuring the expression and activity of p66Shc. The conflicting interpretation of p66Shc's involvement in cellular signalling is due to different experimental designs, particularly the cell lines used. Although p66Shc has been shown to modulate the growth of OC cells, the findings could be highly different depending on the cell lines used in those studies [88]. Manipulation of p66Shc expression has been further associated with opposite effects on cell proliferation, complicating its role in cancer biology [1].

Standardisation of methodologies will increasingly allow for the comparison and reliability of research output. Uniform assay techniques for cellular outcomes such as proliferation and apoptosis are required to evaluate the role of p66Shc [1]. Though the literature suggests that p66Shc modulates ROS levels, the absence of standard measures is a limiting factor in asserting its function. Standardisation of p66Shc expression levels is also crucial for establishing its clinical relevance as a prognostic biomarker. Variable definitions of high and low expression are likely to result in inconsistent clinical prognoses, as various studies on the role of p66Shc in CLL progression have associated its expression with disease outcome [152]. Therefore, there is a need to outline the criteria and techniques to assess the clinical importance of p66Shc in various malignancies.

The institution of standard methodologies will also facilitate the identification of therapeutic targets for p66Shc, mainly in hormone‐sensitive cancers, where steroid hormones partially exert their effects through the action of this protein [85]. Standardisation of the research framework on p66Shc will encourage collaboration among different laboratories and allow better quality and synergy in cancer research. Experimental conditions that vary markedly obscure the proper biological functions of p66Shc and prevent the establishment of valid therapeutic approaches. Some studies support a role for p66Shc in cancer progression, while others propose a pro‐apoptotic role under certain conditions [1]. This apparent duality highlights the importance of adopting stringent and standardised approaches in studying p66Shc, as its role in cancer is determined by both its expression and activity.

### 5.3 Future Research Directions: Exploring p66Shc Interactomes, Post‐Translational Modifications and Its Role in Cancer Stem Cells (CSCs)

Future research should focus on several key areas to further elucidate the role of p66Shc in cancer.

#### 5.3.1 Exploring p66Shc Interactomes

Comprehensive studies of p66Shc interactomes will enhance our understanding of its functional role in tumorigenesis. The combination of co‐immunoprecipitation (co‐IP) and mass spectrometry (MS) has emerged as a valuable method for identifying protein–protein interactions involved in various cellular processes. For example, Betts et al. reported that p66Shc regulates ROS signalling in bovine embryos, linking oxidative stress in cancer cells [208]. Ma et al. showed that p66Shc negatively regulates oncogenic RAS hyperactivation, which modulates the metastatic phenotype of cancer cells [209]. A study by Lebiedzińska‐Arciszewska et al. underlined the potential role of p66Shc in metabolic reprogramming, a hallmark of CSCs characterised by changed metabolic pathways, including those that confer a survival and proliferative advantage [42]. Indeed, Wieckowski et al. emphasised the crucial role of p66Shc, highlighting mitochondrial signalling in the balance between survival and apoptosis in tumour cells [210]. Bhat et al. suggested that p66Shc plays a role as a switch in the cell growth response, which appears particularly important for the dynamics of CSCs [1]. The dynamic character of the interactions in which p66Shc participates necessitates modern proteomics techniques, as suggested by Havugimana et al., which enable a deep characterisation of its interactome [211]. High‐throughput MS can identify new interactors of p66Shc, which may represent novel therapeutic targets and biomarkers for cancer intervention. These studies implicate that p66Shc interacts with primary signalling intermediates in tumour development.

#### 5.3.2 Post‐Translational Modifications (PTMs)

The PTMs of p66Shc, such as phosphorylation, acetylation and ubiquitination, represent important regulatory mechanisms that influence p66Shc, with implications for its function, stability and identification of new targets and intervention strategies [94].

Phosphorylation is crucial for p66Shc translocation to mitochondria and generates ROS through the oxidation of Cytc [108, 173]. Such modification could support the oxidative stress response, which indicates a complex regulatory network influenced by growth factors and cytokines [212]. On the other hand, inhibition of the p66Shc signalling complex through the induction of actin depolymerisation, leading to reduced levels of p66Shc, underlines the accuracy of its regulatory mechanisms [3].

Acetylation, particularly at lysine 81, is counterbalanced by the NAD^+^‐dependent deacetylase SIRT1 and mainly influences p66Shc function in regulating oxidative stress and endothelial dysfunction, especially in diabetic disease [94, 96]. Interestingly, this PTM interrelates with phosphorylation because acetylation can facilitate serine phosphorylation and enhance p66Shc activity [94].

Ubiquitination is another major PTM that generally targets proteins for proteasomal degradation, thus influencing p66Shc stability and, consequently, its activity in signalling pathways that regulate cancer progression [85]. For instance, constitutively active Rac1 enhances p66Shc expression by decreasing its ubiquitination, resulting in its stabilisation and increased tumourigenic signalling [56, 85].

#### 5.3.3 Role in CSCs

p66Shc, a key factor in CSCs, is linked to tumorigenesis, metastasis and the development of therapy resistance. It maintains ROS levels, which promote tumorigenesis through genomic instability and the preservation of CSCs while also mediating cellular damage and apoptosis [55, 205]. Indeed, p66Shc has been identified as a mediator of ROS production, which is required for the proliferation of cancer cells, including PCa, thereby playing a role in maintaining CSC properties [55, 205].

p66Shc also regulates metabolic pathways like glycolysis and oxidative phosphorylation that meet the energy demands of rapidly proliferating CSCs [71], potentially impacting the balance between self‐renewal and differentiation. Notably, inhibiting p66Shc leads to an increase in intermediates of glucose metabolism, indicating that p66Shc may act as a metabolic checkpoint in CSCs [71].

Moreover, p66Shc can interact with the main signalling pathways, including Notch and Wnt, to facilitate the self‐renewal of CSC. Active Wnt signalling has been associated with CSC maintenance, and the control of ROS by p66Shc might influence Wnt activity [1, 213]. Furthermore, its interaction with Notch‐3 also regulates self‐renewal and survival in mammary gland stem/progenitor cells, supporting its role in maintaining the stemness of cancer cells and contributing to tumour heterogeneity [214].

The high expression of p66Shc in invasive cancer cell lines, such as MDA‐MB‐231, suggests its potential as a biomarker for CSCs, which could aid in the development of targeted therapeutic strategies [3]. Targeting p66Shc could make CSCs sensitive to conventional therapies and help overcome resistance mechanisms by employing small molecules or RNA interference strategies that target the ROS signalling [1]. The possibility of personalised therapy will depend on deciphering the role of p66Shc in various cancers to develop CSC‐specific therapies.

## 6 Future Perspectives

Using multi‐omics technologies, such as genomics, proteomics, metabolomics and transcriptomics, offers a comprehensive approach to studying p66Shc in cancer. This integration reveals complex regulatory networks, identifies novel biomarkers and therapeutic targets, and enhances our understanding of tumour biology and patient heterogeneity. Additionally, developing targeted therapies against p66Shc, identifying and characterising small‐molecule inhibitors, optimising delivery mechanisms and exploring combination therapies that integrate p66Shc inhibitors with conventional treatments, such as chemotherapy and immunotherapy, may improve patient outcomes. Successfully translating preclinical findings into clinical applications will be crucial for realising the therapeutic potential of p66Shc. Additionally, designing clinical trials to evaluate p66Shc expression as a biomarker for patient stratification and treatment response will be essential. Establishing the efficacy of p66Shc‐targeted therapies within selected tumour types in which high p66Shc expression has been determined is likely to be informatively related to its clinical relevance. Collaboration between basic researchers and clinical oncologists will be necessary to facilitate this translation.

The role of p66Shc within the TME is another critical area of research. Given its intricate interactions with components such as stroma, immune cells and the extracellular matrix, p66Shc may significantly influence tumour behaviour and treatment responses. How p66Shc modifies these interactions may be targeted for new strategies to improve therapeutic efficacy against the TME. Because p66Shc regulates the properties of CSCs and is implicated in metastasis processes, further studies will help to elucidate these features. The elucidation of the role of p66Shc in maintaining stemness and differentiation at each stage of the metastasis cascade could explain the process of tumour recurrence and clarify the resistance mechanisms.

### 6.1 Pharmacological Modulation of p66Shc

Recent studies have highlighted the potential of pharmacological agents to modulate p66Shc activity, underscoring its dual role in cancer to enhance therapeutic outcomes [139, 140]. Indomethacin's antiproliferative effect is attributed to its direct interaction with p66Shc, which competes with activated EGFR without disrupting the ERK‐binding site. It also inhibits cancer cell proliferation by affecting mitochondrial dynamics or Wnt/β‐catenin signalling [204]. Emerging evidence suggests that indomethacin may also potentiate the antimetastatic effects of certain cancer vaccines, thereby offering a synergistic approach in cancer immunotherapy [204, 215]. Furthermore, the utilisation of nanostructured lipid carriers loaded with indomethacin has demonstrated the potential to enhance its delivery specifically to cancer cells, thereby amplifying its anticancer efficacy [216]. These combination strategies and advanced drug delivery systems collectively hold promise for improving the therapeutic outcomes of indomethacin in oncological applications.

Histone deacetylase (HDAC) inhibitors, such as the novel class I HDAC inhibitors (HDACis), 6a‐g, have been shown to restore p66Shc expression in B cells of CLL, where p66Shc deficiency leads to apoptosis resistance (Figure 3). By inhibiting STAT4‐mediated suppression of p66Shc, this compound enhances p66Shc's pro‐apoptotic function, increasing ROS production and sensitising CLL cells to apoptosis [140]. This suggests HDAC inhibitors could be valuable in cancers where p66Shc's pro‐apoptotic role is suppressed, such as CLL. Additionally, *Dioscorea zingiberensis* (DZ), a traditional Chinese medicine that contains saponins and flavonoids, has been shown to reduce p66Shc expression in high‐fat diet/streptozotocin‐induced diabetic nephropathy (DN) in mice (Figure 3). DZ inhibits the NLRP3 inflammasome and reduces p66Shc‐mediated oxidative stress, thereby ameliorating renal injury and inflammation [139]. Due to the overlap between diabetes and cancer comorbidities, modulation of p66Shc by DZ may have implications for cancers like HCC, where oxidative stress and inflammation are key drivers. These findings highlight the specific modulation of p66Shc by drugs, where enhancing its pro‐apoptotic function or suppressing its oncogenic activity can be tailored to specific diseases [139, 140]. Further exploration of modulating agents for p66Shc, including small‐molecule inhibitors, RNA‐based therapies and natural compounds, is warranted to optimise their specificity and efficacy in clinical conditions.

## 7 Conclusion

The p66Shc is emerging as a key player in cancer biology, and its complex role in regulating cellular signalling and oxidative stress influences tumorigenesis. This review aimed to highlight that p66Shc may behave either as an oncogene or a tumour suppressor, depending on the cancer cell type and cellular process. The complex ways through which p66Shc can interfere with critical signalling pathways, such as PI3K/Akt, MAPK and NF‐κB, underscore its potential as a therapeutic target and biomarker in various types of cancers, including breast, colorectal, lung and melanoma. Several therapeutic strategies for targeting p66Shc have been proposed, including small‐molecule inhibitors and gene editing techniques; however, several limitations exist in translating these observations into clinical settings. Overcoming the limits of the existing literature requires providing standardised approaches and considering broader implications for p66Shc interactomes and post‐translational modifications. Further clarification of the multifaceted role of p66Shc in cancer will open up new avenues for innovative therapeutic strategies, ultimately translating into improved patient outcomes and new frontiers in personalised cancer medicine.

## Author Contributions

**Davood Zaeifi:** writing – original draft (equal), writing – review and editing (equal). **Khadijeh Jamialahmadi:** writing – review and editing (equal). **Gholamreza Karimi:** conceptualization (equal), writing – review and editing (equal).

## Ethics Statement

The authors have nothing to report.

## Consent

The authors have nothing to report.

## Conflicts of Interest

The authors declare no conflicts of interest.

## Data Availability

The authors have nothing to report.

## References

[1] S. S. Bhat , D. Anand , and F. A. Khanday , “p66Shc as a Switch in Bringing About Contrasting Responses in Cell Growth: Implications on Cell Proliferation and Apoptosis,” Molecular Cancer 14 (2015): 76, 10.1186/s12943-015-0354-9. 25890053 PMC4421994

[2] S. F. Sampaio , A. F. Branco , A. Wojtala , I. Vega‐Naredo , M. R. Wieckowski , and P. J. Oliveira , “p66Shc Signaling Is Involved in Stress Responses Elicited by Anthracycline Treatment of Rat Cardiomyoblasts,” Archives of Toxicology 90, no. 7 (2016): 1669–1684, 10.1007/s00204-015-1583-9. 26318906

[3] R. Ali , H. A. Mir , R. Hamid , et al., “Actin Modulation Regulates the Alpha‐1‐Syntrophin/p66Shc Mediated Redox Signaling Contributing to the RhoA GTPase Protein Activation in Breast Cancer Cells,” Frontiers in Oncology 12 (2022): 841303, 10.3389/fonc.2022.841303. 35273919 PMC8904154

[4] R. A. Baba , H. A. Mir , T. A. Mokhdomi , H. F. Bhat , A. Ahmad , and F. A. Khanday , “Quercetin Suppresses ROS Production and Migration by Specifically Targeting Rac1 Activation in Gliomas,” Frontiers in Pharmacology 15 (2024): 1318797, 10.3389/fphar.2024.1318797. 38362155 PMC10867961

[5] H. A. Mir , R. Ali , U. Mushtaq , and F. A. Khanday , “Structure‐Functional Implications of Longevity Protein p66Shc in Health and Disease,” Ageing Research Reviews 63 (2020): 101139, 10.1016/j.arr.2020.101139. 32795504

[6] L. Haslem , J. M. Hays , and F. A. Hays , “p66Shc in Cardiovascular Pathology,” Cells 11, no. 11 (2022): 1855, 10.3390/cells11111855. 35681549 PMC9180016

[7] S. Kumar , “P66Shc and Vascular Endothelial Function,” Bioscience Reports 39, no. 4 (2019): BSR20182134, 10.1042/BSR20182134. 30918103 PMC6488855

[8] M. Rajendran , P. Thomes , L. Zhang , S. Veeramani , and M. F. Lin , “p66Shc—A Longevity Redox Protein in Human Prostate Cancer Progression and Metastasis: p66Shc in Cancer Progression and Metastasis,” Cancer Metastasis Reviews 29, no. 1 (2010): 207–222, 10.1007/s10555-010-9213-8. 20111892 PMC2909029

[9] Z. J. Han , Y. H. Feng , B. H. Gu , Y. M. Li , and H. Chen , “The Post‐Translational Modification, SUMOylation, and Cancer (Review),” International Journal of Oncology 52, no. 4 (2018): 1081–1094, 10.3892/ijo.2018.4280. 29484374 PMC5843405

[10] A. Glaviano , A. S. C. Foo , H. Y. Lam , et al., “PI3K/AKT/mTOR Signaling Transduction Pathway and Targeted Therapies in Cancer,” Molecular Cancer 22, no. 1 (2023): 138, 10.1186/s12943-023-01827-6. 37596643 PMC10436543

[11] A. B. S. Khorasani , N. Hafezi , M.‐J. Sanaei , F. Jafari‐Raddani , A. Pourbagheri‐Sigaroodi , and D. Bashash , “The PI3K/AKT/mTOR Signaling Pathway in Breast Cancer: Review of Clinical Trials and Latest Advances,” Cell Biochemistry and Function 42, no. 3 (2024): e3998, 10.1002/cbf.3998. 38561964

[12] A. A. Tomilov , V. Bicocca , R. A. Schoenfeld , et al., “Decreased Superoxide Production in Macrophages of Long‐Lived p66Shc Knock‐Out Mice,” Journal of Biological Chemistry 285, no. 2 (2010): 1153–1165, 10.1074/jbc.M109.017491. 19892704 PMC2801244

[13] B. Liu , Y. Liu , L. Zhao , et al., “Upregulation of microRNA‐135b and microRNA‐182 Promotes Chemoresistance of Colorectal Cancer by Targeting ST6GALNAC2 via PI3K/AKT Pathway,” Molecular Carcinogenesis 56, no. 12 (2017): 2669–2680, 10.1002/mc.22710. 28767179

[14] H. L. Robbins and A. Hague , “The PI3K/Akt Pathway in Tumors of Endocrine Tissues,” Frontiers in Endocrinology 6 (2015): 188, 10.3389/fendo.2015.00188. 26793165 PMC4707207

[15] O. Soriano , M. Alcon‐Perez , M. Vicente‐Manzanares , and E. Castellano , “The Crossroads Between RAS and RHO Signaling Pathways in Cellular Transformation, Motility and Contraction,” Genes 12, no. 6 (2021): 819, 10.3390/genes12060819. 34071831 PMC8229961

[16] R. Roskoski, Jr. , “Properties of FDA‐Approved Small Molecule Phosphatidylinositol 3‐Kinase Inhibitors Prescribed for the Treatment of Malignancies,” Pharmacological Research 168 (2021): 105579, 10.1016/j.phrs.2021.105579. 33774181

[17] A. C. da C Pinaffi‐Langley , E. Melia , and F. A. Hays , “Exploring the Gut‐Mitochondrial Axis: p66Shc Adapter Protein and Its Implications for Metabolic Disorders,” International Journal of Molecular Sciences 25, no. 7 (2024): 3656, 10.3390/ijms25073656. 38612468 PMC11011581

[18] Z. Wang , “Regulation of Cell Cycle Progression by Growth Factor‐Induced Cell Signaling,” Cells 10, no. 12 (2021): 3327, 10.3390/cells10123327. 34943835 PMC8699227

[19] Y. C. Chang , M. H. Chan , Y. F. Yang , C. H. Li , and M. Hsiao , “Glucose Transporter 4: Insulin Response Mastermind, Glycolysis Catalyst and Treatment Direction for Cancer Progression,” Cancer Letters 563 (2023): 216179, 10.1016/j.canlet.2023.216179. 37061122

[20] J. Li , J. Wang , D. Xie , et al., “Characteristics of the PI3K/AKT and MAPK/ERK Pathways Involved in the Maintenance of Self‐Renewal in Lung Cancer Stem‐Like Cells,” International Journal of Biological Sciences 17, no. 5 (2021): 1191–1202, 10.7150/ijbs.57871. 33867839 PMC8040472

[21] Y. Liu , F. Lin , Y. Chen , et al., “Cryptotanshinone Inhibites Bladder Cancer Cell Proliferation and Promotes Apoptosis via the PTEN/PI3K/AKT Pathway,” Journal of Cancer 11, no. 2 (2020): 488–499, 10.7150/jca.31422. 31897244 PMC6930428

[22] Y. Mao , L. Xi , Q. Li , et al., “Regulation of Cell Apoptosis and Proliferation in Pancreatic Cancer Through PI3K/Akt Pathway via Polo‐Like Kinase 1,” Oncology Reports 36, no. 1 (2016): 49–56, 10.3892/or.2016.4820. 27220401 PMC4899032

[23] C. Braicu , M. Buse , C. Busuioc , et al., “A Comprehensive Review on MAPK: A Promising Therapeutic Target in Cancer,” Cancers (Basel) 11, no. 10 (2019): 1618, 10.3390/cancers11101618. 31652660 PMC6827047

[24] S. M. Weber , N. M. Brossier , A. Prechtl , et al., “R‐Ras Subfamily Proteins Elicit Distinct Physiologic Effects and Phosphoproteome Alterations in Neurofibromin‐Null MPNST Cells,” Cell Communication and Signaling: CCS 19, no. 1 (2021): 95, 10.1186/s12964-021-00773-4. 34530870 PMC8447793

[25] Z. Moghadamchargari , M. Shirzadeh , C. Liu , et al., “Molecular Assemblies of the Catalytic Domain of SOS With KRas and Oncogenic Mutants,” Proceedings of the National Academy of Sciences of the United States of America 118, no. 12 (2021): e2022403118, 10.1073/pnas.2022403118. 33723061 PMC8000204

[26] E. Podyacheva , J. Snezhkova , A. Onopchenko , V. Dyachuk , and Y. Toropova , “The Role of MicroRNAs in the Pathogenesis of Doxorubicin‐Induced Vascular Remodeling,” International Journal of Molecular Sciences 25, no. 24 (2024): 13335, 10.3390/ijms252413335. 39769102 PMC11728060

[27] C. A. Bill , C. M. Allen , and C. M. Vines , “CC Chemokine Receptor 7 in Cancer,” Cells 11, no. 4 (2022): 656, 10.3390/cells11040656. 35203305 PMC8870371

[28] H. Yan , L. He , D. Lv , J. Yang , and Z. Yuan , “The Role of the Dysregulated JNK Signaling Pathway in the Pathogenesis of Human Diseases and Its Potential Therapeutic Strategies: A Comprehensive Review,” Biomolecules 14, no. 2 (2024): 243, 10.3390/biom14020243. 38397480 PMC10887252

[29] O. Fedorova , S. Parfenyev , A. Daks , O. Shuvalov , and N. A. Barlev , “The Role of PTEN in Epithelial‐Mesenchymal Transition,” Cancers 14, no. 15 (2022): 3786, 10.3390/cancers14153786. 35954450 PMC9367281

[30] M. A. Yapryntseva , B. Zhivotovsky , and V. Gogvadze , “Permeabilization of the Outer Mitochondrial Membrane: Mechanisms and Consequences,” Biochimica et Biophysica Acta—Molecular Basis of Disease 1870, no. 7 (2024): 167317, 10.1016/j.bbadis.2024.167317. 38909847

[31] S. Kaushal , S. Gupta , S. Shefrin , et al., “Synthetic and Natural Inhibitors of Mortalin for Cancer Therapy,” Cancers 16, no. 20 (2024): 3470, 10.3390/cancers16203470. 39456564 PMC11506508

[32] H. Javid , P. Hashemian , S. Yazdani , A. Sharbaf Mashhad , and M. Karimi‐Shahri , “The Role of Heat Shock Proteins in Metastatic Colorectal Cancer: A Review,” Journal of Cellular Biochemistry 123, no. 11 (2022): 1704–1735, 10.1002/jcb.30326. 36063530

[33] A. Zannini , A. Rustighi , E. Campaner , and G. Del Sal , “Oncogenic Hijacking of the PIN1 Signaling Network,” Frontiers in Oncology 9 (2019): 94, 10.3389/fonc.2019.00094. 30873382 PMC6401644

[34] H.‐H. Chuang , Y.‐Y. Zhen , Y.‐C. Tsai , et al., “Targeting Pin1 for Modulation of Cell Motility and Cancer Therapy,” Biomedicine 9, no. 4 (2021): 359, 10.3390/biomedicines9040359. PMC8065645 33807199

[35] B. Vurusaner , G. Poli , and H. Basaga , “Tumor Suppressor Genes and ROS: Complex Networks of Interactions,” Free Radical Biology and Medicine 52, no. 1 (2012): 7–18, 10.1016/j.freeradbiomed.2011.09.035. 22019631

[36] Y. S. Hori , A. Kuno , R. Hosoda , and Y. Horio , “Regulation of FOXOs and p53 by SIRT1 Modulators Under Oxidative Stress,” PLoS One 8, no. 9 (2013): e73875, 10.1371/journal.pone.0073875. 24040102 PMC3770600

[37] U. Degirmenci , M. Wang , and J. Hu , “Targeting Aberrant RAS/RAF/MEK/ERK Signaling for Cancer Therapy,” Cells 9, no. 1 (2020): 198, 10.3390/cells9010198. 31941155 PMC7017232

[38] A. Chahdi and A. Sorokin , “Endothelin‐1 Induces p66Shc Activation Through EGF Receptor Transactivation: Role of Beta(1)Pix/Galpha(i3) Interaction,” Cellular Signalling 22, no. 2 (2010): 325–329, 10.1016/j.cellsig.2009.09.039. 19804820 PMC2788086

[39] N. A. Edwards , A. J. Watson , and D. H. Betts , “Knockdown of p66Shc Alters Lineage‐Associated Transcription Factor Expression in Mouse Blastocysts,” Stem Cells and Development 27, no. 21 (2018): 1479–1493, 10.1089/scd.2018.0131. 30091687 PMC6209429

[40] S. Barangi , P. Hosseinzadeh , G. Karimi , Z. Tayarani Najaran , and S. Mehri , “Osthole Attenuated Cytotoxicity Induced by 6‐OHDA in SH‐SY5Y Cells Through Inhibition of JAK/STAT and MAPK Pathways,” Iranian Journal of Basic Medical Sciences 26, no. 8 (2023): 953–959, 10.22038/IJBMS.2023.68292.14905. 37427324 PMC10329246

[41] J. Aseervatham , “Cytoskeletal Remodeling in Cancer,” Biology (Basel) 9, no. 11 (2020): 385, 10.3390/biology9110385. 33171868 PMC7695181

[42] M. Lebiedzinska‐Arciszewska , M. Oparka , I. Vega‐Naredo , et al., “The Interplay Between p66Shc, Reactive Oxygen Species and Cancer Cell Metabolism,” European Journal of Clinical Investigation 45, no. Suppl 1 (2015): 25–31, 10.1111/eci.12364. 25524583

[43] L. Sun , L. Xiao , J. Nie , et al., “p66Shc Mediates High‐Glucose and Angiotensin II‐Induced Oxidative Stress Renal Tubular Injury via Mitochondrial‐Dependent Apoptotic Pathway,” American Journal of Physiology. Renal Physiology 299, no. 5 (2010): F1014–F1025, 10.1152/ajprenal.00414.2010. 20739391 PMC2980400

[44] A. Natalicchio , F. Tortosa , S. Perrini , L. Laviola , and F. Giorgino , “p66Shc, a Multifaceted Protein Linking Erk Signalling, Glucose Metabolism, and Oxidative Stress,” Archives of Physiology and Biochemistry 117, no. 3 (2011): 116–124, 10.3109/13813455.2011.562513. 21506908

[45] X. Chen , H. Li , H. Cheng , et al., “Vitexin Attenuates Smoke Inhalation Induced Acute Lung Injury in Rats by Inhibiting Oxidative Stress via PKC β/p66Shc Signaling Pathway,” Tropical Journal of Pharmaceutical Research 21, no. 12 (2022): 2507–2518, 10.4314/tjpr.v21i12.2.

[46] S. Mousavi , M. A. Khazeei Tabari , A. Bagheri , N. Samieefar , N. Shaterian , and R. Kelishadi , “The Role of p66Shc in Diabetes: A Comprehensive Review From Bench to Bedside,” Journal Diabetes Research 2022 (2022): 7703520, 10.1155/2022/7703520. PMC9715346 36465704

[47] T. He , H. Shen , S. Wang , et al., “MicroRNA‐3613‐5p Promotes Lung Adenocarcinoma Cell Proliferation Through a RELA and AKT/MAPK Positive Feedback Loop,” Molecular Therapy—Nucleic Acids 22 (2020): 572–583, 10.1016/j.omtn.2020.09.024. 33230458 PMC7562961

[48] J. A. Prescott , J. P. Mitchell , and S. J. Cook , “Inhibitory Feedback Control of NF‐kappaB Signalling in Health and Disease,” Biochemical Journal 478, no. 13 (2021): 2619–2664, 10.1042/BCJ20210139. 34269817 PMC8286839

[49] S. C. Gupta , N. Awasthee , V. Rai , S. Chava , V. Gunda , and K. B. Challagundla , “Long Non‐Coding RNAs and Nuclear Factor‐kappaB Crosstalk in Cancer and Other Human Diseases,” Biochimica et Biophysica Acta (BBA)‐Reviews on Cancer 1873, no. 1 (2020): 188316, 10.1016/j.bbcan.2019.188316. 31639408 PMC7775411

[50] S. Mahdiani , N. Omidkhoda , S. Heidari , A. W. Hayes , and G. Karimi , “Protective Effect of Luteolin Against Chemical and Natural Toxicants by Targeting NF‐kappaB Pathway,” BioFactors 48, no. 4 (2022): 744–762, 10.1002/biof.1876. 35861671

[51] A. Rosell , A. A. Krygowska , M. A. Pérez , et al., “RAS‐p110α Signalling in Macrophages Is Required for Effective Inflammatory Response and Resolution of Inflammation,” eLife 13 (2025): RP94590, 10.7554/elife.94590.3. 40272400 PMC12021417

[52] L. Lopresti , N. Capitani , V. Tatangelo , et al., “p66Shc Deficiency in CLL Cells Enhances PD‐L1 Expression and Suppresses Immune Synapse Formation,” Frontiers in Cell and Development Biology 12 (2024): 1297116, 10.3389/fcell.2024.1297116. PMC10883382 38389706

[53] V. Tatangelo , G. Boncompagni , N. Capitani , et al., “p66Shc Deficiency in Chronic Lymphocytic Leukemia Promotes Chemokine Receptor Expression Through the ROS‐Dependent Inhibition of NF‐κB,” Frontiers in Oncology 12 (2022): 877495, 10.3389/fonc.2022.877495. 35847884 PMC9278989

[54] Z. Li , X. Li , X. Du , et al., “The Interaction Between lncRNA SNHG1 and miR‐140 in Regulating Growth and Tumorigenesis via the TLR4/NF‐kappaB Pathway in Cholangiocarcinoma,” Oncology Research 27, no. 6 (2019): 663–672, 10.3727/096504018X15420741307616. 30764893 PMC7848332

[55] M. A. Ingersoll , Y. W. Chou , J. S. Lin , et al., “p66Shc Regulates Migration of Castration‐Resistant Prostate Cancer Cells,” Cellular Signalling 46 (2018): 1–14, 10.1016/j.cellsig.2018.02.008. 29462661 PMC5882563

[56] M. Bashir , D. Kirmani , H. F. Bhat , et al., “P66shc and Its Downstream Eps8 and Rac1 Proteins Are Upregulated in Esophageal Cancers,” Cell Communication and Signaling: CCS 8 (2010): 13, 10.1186/1478-811X-8-13. 20565814 PMC2901305

[57] M. Devanaboyina , J. Kaur , E. Whiteley , et al., “NF‐kappaB Signaling in Tumor Pathways Focusing on Breast and Ovarian Cancer,” Oncology Reviews 16 (2022): 10568, 10.3389/or.2022.10568. 36531159 PMC9756851

[58] F. Hakuno and S. I. Takahashi , “IGF1 Receptor Signaling Pathways,” Journal of Molecular Endocrinology 61, no. 1 (2018): T69–T86, 10.1530/JME-17-0311. 29535161

[59] K. D. Wright , A. Staruschenko , and A. Sorokin , “Role of Adaptor Protein p66Shc in Renal Pathologies,” American Journal of Physiology. Renal Physiology 314, no. 2 (2018): F143–F153, 10.1152/ajprenal.00414.2017. 28978535 PMC5866456

[60] Z. Zhang , L. Yao , J. Yang , Z. Wang , and G. Du , “PI3K/Akt and HIF‐1 Signaling Pathway in Hypoxia‐Ischemia (Review),” Molecular Medicine Reports 18, no. 4 (2018): 3547–3554, 10.3892/mmr.2018.9375. 30106145 PMC6131612

[61] R. Shakeri , A. Kheirollahi , and J. Davoodi , “Apaf‐1: Regulation and Function in Cell Death,” Biochimie 135 (2017): 111–125, 10.1016/j.biochi.2017.02.001. 28192157

[62] S. K. Sahu , C. Kaur , A. Yadav , et al., “Caspase 7 Mutations and Their Activators,” in Caspases as Molecular Targets for Cancer Therapy, ed. A. Vaidya (Academic Press, 2024), 105–130, 10.1016/B978-0-443-15644-1.00006-7.

[63] D. Kumar and M. I. Hassan , “Recent Advances in the Therapeutic Development of ERK Inhibitors,” in Protein Kinase Inhibitors, ed. M. I. Hassan and S. Noor (Academic Press, 2022), 129–178, 10.1016/B978-0-323-91287-7.00023-5.

[64] G. S. Bhavani and A. Palanisamy , “SNAIL Driven by a Feed Forward Loop Motif Promotes TGF‐β Induced Epithelial to Mesenchymal Transition,” Biomedical Physics & Engineering Express 8, no. 4 (2022): 5012, 10.1088/2057-1976/ac7896. 35700712

[65] H. Y. K. Yip , S. Y. Shin , A. Chee , et al., “Integrative Modeling Uncovers p21‐Driven Drug Resistance and Prioritizes Therapies for PIK3CA‐Mutant Breast Cancer,” npj Precision Oncology 8, no. 1 (2024): 20, 10.1038/s41698-024-00496-y. 38273040 PMC10810864

[66] L. Avila‐Carrasco , P. Majano , J. A. Sanchez‐Tomero , et al., “Natural Plants Compounds as Modulators of Epithelial‐To‐Mesenchymal Transition,” Frontiers in Pharmacology 10 (2019): 715, 10.3389/fphar.2019.00715. 31417401 PMC6682706

[67] S. Usman , N. H. Waseem , T. K. N. Nguyen , et al., “Vimentin Is at the Heart of Epithelial Mesenchymal Transition (EMT) Mediated Metastasis,” Cancers (Basel) 13, no. 19 (2021): 4985, 10.3390/cancers13194985. 34638469 PMC8507690

[68] I. Malami , A. Muhammad , A. P. Yusuf , and B. S. Katsayal , “AMPK Signaling in Cancer,” in Handbook of Cancer and Immunology, ed. N. Rezaei (Springer Nature Switzerland, 2025), 1–22, 10.1007/978-3-030-80962-1_398-1.

[69] V. Pourbarkhordar , S. Rahmani , A. Roohbakhsh , A. W. Hayes , and G. Karimi , “Melatonin Effect on Breast and Ovarian Cancers by Targeting the PI3K/Akt/mTOR Pathway,” IUBMB Life 76, no. 12 (2024): 1035–1049, 10.1002/iub.2900. 39212097

[70] A. Engin , “Protein Kinases in Obesity, and the Kinase‐Targeted Therapy,” in Obesity and Lipotoxicity, vol. 1460 (Springer, 2024), 199–229, 10.1007/978-3-031-63657-8_7. 39287853

[71] M. A. Soliman , A. M. Abdel Rahman , D. W. Lamming , et al., “The Adaptor Protein p66Shc Inhibits mTOR‐Dependent Anabolic Metabolism,” Science Signaling 7, no. 313 (2014): ra17, 10.1126/scisignal.2004785. 24550542 PMC4260967

[72] J. Zuo , Z. Zhang , M. Li , et al., “The Crosstalk Between Reactive Oxygen Species and Noncoding RNAs: From Cancer Code to Drug Role,” Molecular Cancer 21, no. 1 (2022): 30, 10.1186/s12943-021-01488-3. 35081965 PMC8790843

[73] F. Yarmohammadi , Z. Ebrahimian , and G. Karimi , “MicroRNAs Target the PI3K/Akt/p53 and the Sirt1/Nrf2 Signaling Pathways in Doxorubicin‐Induced Cardiotoxicity,” Journal of Biochemical and Molecular Toxicology 37, no. 2 (2023): e23261, 10.1002/jbt.23261. 36416353

[74] Q. Li , Y.‐R. Kim , A. Vikram , et al., “P66Shc‐Induced microRNA‐34a Causes Diabetic Endothelial Dysfunction by Downregulating Sirtuin1,” Arteriosclerosis, Thrombosis, and Vascular Biology 36, no. 12 (2016): 2394–2403, 10.1161/ATVBAHA.116.308321. 27789474 PMC5293179

[75] V. H. Trinh , T. Nguyen Huu , D. K. Sah , et al., “Redox Regulation of PTEN by Reactive Oxygen Species: Its Role in Physiological Processes,” Antioxidants (Basel) 13, no. 2 (2024): 199, 10.3390/antiox13020199. 38397797 PMC10886030

[76] S. Barangi , A. W. Hayes , and G. Karimi , “The Role of lncRNAs/miRNAs/Sirt1 Axis in Myocardial and Cerebral Injury,” Cell Cycle 22, no. 9 (2023): 1062–1073, 10.1080/15384101.2023.2172265. 36703306 PMC10081082

[77] Q. Lu , H. Feng , H. Chen , et al., “Role of DCLK1 in Oncogenic Signaling,” International Journal of Oncology 61, no. 5 (2022): 137, 10.3892/ijo.2022.5427. 36148883

[78] A. La Colla , A. Vasconsuelo , L. Milanesi , and L. Pronsato , “17β‐Estradiol Protects Skeletal Myoblasts From Apoptosis Through p53, Bcl‐2, and FoxO Families,” Journal of Cellular Biochemistry 118, no. 1 (2017): 104–115, 10.1002/jcb.25616. 27249370

[79] V. Villarreal‐García , J. R. Estupiñan‐Jiménez , P. E. Vivas‐Mejía , V. Gonzalez‐Villasana , J. M. Vázquez‐Guillén , and D. Reséndez‐Pérez , “A Vicious Circle in Breast Cancer: The Interplay Between Inflammation, Reactive Oxygen Species, and microRNAs,” Frontiers in Oncology 12 (2022): 980694, 10.3389/fonc.2022.980694. 36226048 PMC9548555

[80] S. Koontongkaew , “The Tumor Microenvironment Contribution to Development, Growth, Invasion and Metastasis of Head and Neck Squamous Cell Carcinomas,” Journal of Cancer 4, no. 1 (2013): 66–83, 10.7150/jca.5112. 23386906 PMC3564248

[81] J. Xu , M. Kitada , and D. Koya , “The Impact of Mitochondrial Quality Control by Sirtuins on the Treatment of Type 2 Diabetes and Diabetic Kidney Disease,” Biochimica et Biophysica Acta (BBA)—Molecular Basis of Disease 1866, no. 6 (2020): 165756, 10.1016/j.bbadis.2020.165756. 32147421

[82] M. Landry , V. Pomerleau , and C. Saucier , “Non‐Canonical Dynamic Mechanisms of Interaction Between the p66Shc Protein and Met Receptor,” Biochemical Journal 473, no. 11 (2016): 1617–1627, 10.1042/BCJ20160249. 27048591 PMC4888465

[83] J. Chen , G. Gao , L. Li , et al., “Pan‐Cancer Study of SHC‐Adaptor Protein 1 (SHC1) as a Diagnostic, Prognostic and Immunological Biomarker in Human Cancer,” Frontiers in Genetics 13 (2022): 817118, 10.3389/fgene.2022.817118. 35601500 PMC9115805

[84] J. Hudson , J. R. Ha , V. Sabourin , et al., “p66ShcA Promotes Breast Cancer Plasticity by Inducing an Epithelial‐To‐Mesenchymal Transition,” Molecular and Cellular Biology 34, no. 19 (2014): 3689–3701, 10.1128/MCB.00341-14. 25071152 PMC4187732

[85] S. Kumar , S. Kumar , M. Rajendran , et al., “Steroids Up‐Regulate p66Shc Longevity Protein in Growth Regulation by Inhibiting Its Ubiquitination,” PLoS One 6, no. 1 (2011): e15942, 10.1371/journal.pone.0015942. 21264241 PMC3021521

[86] A. Onnis , V. Cianfanelli , C. Cassioli , et al., “The Pro‐Oxidant Adaptor p66SHC Promotes B Cell Mitophagy by Disrupting Mitochondrial Integrity and Recruiting LC3‐II,” Autophagy 14, no. 12 (2018): 2117–2138, 10.1080/15548627.2018.1505153. 30109811 PMC6984773

[87] S. Fasolato , M. Ruvoletto , G. Nardo , et al., “Low P66shc With High SerpinB3 Levels Favors Necroptosis and Better Survival in Hepatocellular Carcinoma,” Biology (Basel) 10, no. 5 (2021): 363, 10.3390/biology10050363. 33922660 PMC8145214

[88] S. Muniyan , Y. W. Chou , T. J. Tsai , et al., “p66Shc Longevity Protein Regulates the Proliferation of Human Ovarian Cancer Cells,” Molecular Carcinogenesis 54, no. 8 (2015): 618–631, 10.1002/mc.22129. 24395385 PMC4117819

[89] S. Modi , W. Jacot , T. Yamashita , et al., “Trastuzumab Deruxtecan in Previously Treated HER2‐Low Advanced Breast Cancer,” New England Journal of Medicine 387, no. 1 (2022): 9–20, 10.1056/NEJMoa2203690. 35665782 PMC10561652

[90] J. K. Gunther , A. Nikolajevic , S. Ebner , J. Troppmair , and S. Khalid , “Rigosertib‐Activated JNK1/2 Eliminate Tumor Cells Through p66Shc Activation,” Biology 9, no. 5 (2020): 99, 10.3390/biology9050099. 32429320 PMC7284707

[91] M. Miyazawa and Y. Tsuji , “Evidence for a Novel Antioxidant Function and Isoform‐Specific Regulation of the Human p66Shc Gene,” Molecular Biology of the Cell 25, no. 13 (2014): 2116–2127, 10.1091/mbc.E13-11-0666. 24807908 PMC4072584

[92] N. T. Bain , P. Madan , and D. H. Betts , “Elevated p66Shc Is Associated With Intracellular Redox Imbalance in Developmentally Compromised Bovine Embryos,” Molecular Reproduction and Development 80, no. 1 (2013): 22–34, 10.1002/mrd.22128. 23109234

[93] Y. Zhao , Z. Wang , D. Feng , et al., “p66Shc Contributes to Liver Fibrosis Through the Regulation of Mitochondrial Reactive Oxygen Species,” Theranostics 9, no. 5 (2019): 1510–1522, 10.7150/thno.29620. 30867846 PMC6401497

[94] S. Kumar , Y. R. Kim , A. Vikram , et al., “Sirtuin1‐Regulated Lysine Acetylation of p66Shc Governs Diabetes‐Induced Vascular Oxidative Stress and Endothelial Dysfunction,” Proceedings of the National Academy of Sciences 114, no. 7 (2017): 1714–1719, 10.1073/pnas.1614112114. PMC5321021 28137876

[95] R. Fu , J. Zhou , R. Wang , et al., “Protocatechuic Acid‐Mediated miR‐219a‐5p Activation Inhibits the p66shc Oxidant Pathway to Alleviate Alcoholic Liver Injury,” Oxidative Medicine and Cellular Longevity 2019 (2019): 3527809, 10.1155/2019/3527809. 31428222 PMC6683775

[96] M. Mishra , A. J. Duraisamy , S. Bhattacharjee , and R. A. Kowluru , “Adaptor Protein p66Shc: A Link Between Cytosolic and Mitochondrial Dysfunction in the Development of Diabetic Retinopathy,” Antioxidants & Redox Signaling 30, no. 13 (2019): 1621–1634, 10.1089/ars.2018.7542. 30105917 PMC6459280

[97] M. Hashimoto , T. Kobayashi , H. Tashiro , K. Arihiro , A. Kikuchi , and H. Ohdan , “H‐Prune Is Associated With Poor Prognosis and Epithelial‐Mesenchymal Transition in Patients With Colorectal Liver Metastases,” International Journal of Cancer 139, no. 4 (2016): 812–823, 10.1002/ijc.30118. 27037526

[98] J.‐G. He , L. Li , Y. Qin , W. Yu , X. He , and R. Gang , “Aurora‐A Regulates Progression and Metastasis of Colorectal Cancer by Promoting Slug Activity,” Technology in Cancer Research & Treatment 16, no. 6 (2017): 766–775, 10.1177/1533034616682172.

[99] Z. Wang , Y. Liu , L. Lu , et al., “Fibrillin‐1, Induced by Aurora‐A but Inhibited by BRCA2, Promotes Ovarian Cancer Metastasis,” Oncotarget 6, no. 9 (2015): 6670–6683, 10.18632/oncotarget.3118. 25749384 PMC4466642

[100] I. R. Oh , B. Raymundo , M. Kim , and C. W. Kim , “Mesenchymal Stem Cells Co‐Cultured With Colorectal Cancer Cells Showed Increased Invasive and Proliferative Abilities due to Its Altered p53/TGF‐β1 Levels,” Bioscience, Biotechnology, and Biochemistry 84, no. 2 (2020): 256–267, 10.1080/09168451.2019.1676692. 31601153

[101] M. Lacroix , R. Riscal , G. Arena , L. K. Linares , and L. Le Cam , “Metabolic Functions of the Tumor Suppressor p53: Implications in Normal Physiology, Metabolic Disorders, and Cancer,” Molecular Metabolism 33 (2020): 2–22, 10.1016/j.molmet.2019.10.002. 31685430 PMC7056927

[102] G. Cui , Z. Wang , H. Liu , and Z. Pang , “Cytokine‐Mediated Crosstalk Between Cancer Stem Cells and Their Inflammatory Niche From the Colorectal Precancerous Adenoma Stage to the Cancerous Stage: Mechanisms and Clinical Implications,” Frontiers in Immunology 13 (2022): 1057181, 10.3389/fimmu.2022.1057181. 36466926 PMC9714270

[103] D. D. Lin , Y. Shen , S. Qiao , et al., “Upregulation of OTUD7B (Cezanne) Promotes Tumor Progression via AKT/VEGF Pathway in Lung Squamous Carcinoma and Adenocarcinoma,” Frontiers in Oncology 9 (2019): 862, 10.3389/fonc.2019.00862. 31572671 PMC6749047

[104] Y. Yang , Z. Li , H. Yuan , et al., “Reciprocal Regulatory Mechanism Between miR‐214‐3p and FGFR1 in FGFR1‐Amplified Lung Cancer,” Oncogene 8, no. 9 (2019): 50, 10.1038/s41389-019-0151-1. PMC6731303 31492847

[105] E. Riquelme , C. Behrens , H. Y. Lin , et al., “Modulation of EZH2 Expression by MEK‐ERK or PI3K‐AKT Signaling in Lung Cancer Is Dictated by Different KRAS Oncogene Mutations,” Cancer Research 76, no. 3 (2016): 675–685, 10.1158/0008-5472.CAN-15-1141. 26676756 PMC4738155

[106] Y. C. Wang , D. W. Wu , T. C. Wu , L. Wang , C. Y. Chen , and H. Lee , “Dioscin Overcome TKI Resistance in EGFR‐Mutated Lung Adenocarcinoma Cells via Down‐Regulation of Tyrosine Phosphatase SHP2 Expression,” International Journal of Biological Sciences 14, no. 1 (2018): 47–56, 10.7150/ijbs.22209. 29483824 PMC5821048

[107] I. Arany , J. Clark , D. K. Reed , and L. A. Juncos , “Chronic Nicotine Exposure Augments Renal Oxidative Stress and Injury Through Transcriptional Activation of p66shc,” Nephrology, Dialysis, Transplantation 28, no. 6 (2013): 1417–1425, 10.1093/ndt/gfs596. PMC3685305 23328708

[108] I. Arany , A. Faisal , J. S. Clark , T. Vera , R. Baliga , and Y. Nagamine , “p66SHC‐Mediated Mitochondrial Dysfunction in Renal Proximal Tubule Cells During Oxidative Injury,” American Journal of Physiology. Renal Physiology 298, no. 5 (2010): F1214–F1221, 10.1152/ajprenal.00639.2009. 20053790

[109] A. Borkowska , A. Sielicka‐Dudzin , A. Herman‐Antosiewicz , et al., “Diallyl Trisulfide‐Induced Prostate Cancer Cell Death Is Associated With Akt/PKB Dephosphorylation Mediated by P‐p66shc,” European Journal of Nutrition 51, no. 7 (2012): 817–825, 10.1007/s00394-011-0260-x. 22020565 PMC3456917

[110] L. Ehsani , C. Cohen , K. E. Fisher , and M. T. Siddiqui , “BRAF Mutations in Metastatic Malignant Melanoma: Comparison of Molecular Analysis and Immunohistochemical Expression,” Applied Immunohistochemistry & Molecular Morphology 22, no. 9 (2014): 648–651, 10.1097/PAI.0000000000000013. 25046227

[111] A. M. Menzies , L. E. Haydu , L. Visintin , et al., “Distinguishing Clinicopathologic Features of Patients With V600E and V600K BRAF‐Mutant Metastatic Melanoma,” Clinical Cancer Research 18, no. 12 (2012): 3242–3249, 10.1158/1078-0432.CCR-12-0052. 22535154

[112] T. Furlan , S. Khalid , A. V. Nguyen , J. Gunther , and J. Troppmair , “The Oxidoreductase p66Shc Acts as Tumor Suppressor in BRAFV600E‐Transformed Cells,” Molecular Oncology 12, no. 6 (2018): 869–882, 10.1002/1878-0261.12199. 29624862 PMC5983121

[113] L. Liang and Z. Zhang , “Gambogic Acid Inhibits Malignant Melanoma Cell Proliferation Through Mitochondrial p66shc/ROS‐p53/Bax‐Mediated Apoptosis,” Cellular Physiology and Biochemistry 38, no. 4 (2016): 1618–1630, 10.1159/000443102. 27119348

[114] Y. Yu , D. Ladeiras , Y. Xiong , et al., “Arginase‐II Promotes Melanoma Migration and Adhesion Through Enhancing Hydrogen Peroxide Production and STAT3 Signaling,” Journal of Cellular Physiology 235, no. 12 (2020): 9997–10011, 10.1002/jcp.29814. 32468644

[115] U. Mushtaq , M. Bashir , S. Nabi , and F. A. Khanday , “Epidermal Growth Factor Receptor and Integrins Meet Redox Signaling Through P66shc and Rac1,” Cytokine 146 (2021): 155625, 10.1016/j.cyto.2021.155625. 34157521

[116] B. Puente‐Cobacho , A. Varela‐López , J. L. Quiles , and L. Vera‐Ramirez , “Involvement of Redox Signalling in Tumour Cell Dormancy and Metastasis,” Cancer Metastasis Reviews 42, no. 1 (2023): 49–85, 10.1007/s10555-022-10077-9. 36701089 PMC10014738

[117] M. Dhavale , M. K. Abdelaal , A. Alam , et al., “Androgen Receptor Signaling and the Emergence of Lethal Neuroendocrine Prostate Cancer With the Treatment‐Induced Suppression of the Androgen Receptor: A Literature Review,” Cureus 13, no. 2 (2021): e13402, 10.7759/cureus.13402. 33754118 PMC7971732

[118] T. Zhou and Q. Feng , “Androgen Receptor Signaling and Spatial Chromatin Organization in Castration‐Resistant Prostate Cancer,” Frontiers in Medicine 9 (2022): 924087, 10.3389/fmed.2022.924087. 35966880 PMC9372301

[119] M. Ehsani , F. O. David , and A. Baniahmad , “Androgen Receptor‐Dependent Mechanisms Mediating Drug Resistance in Prostate Cancer,” Cancers (Basel) 13, no. 7 (2021): 1534, 10.3390/cancers13071534. 33810413 PMC8037957

[120] S. V. Bhagwat , W. T. McMillen , S. Cai , et al., “ERK Inhibitor LY3214996 Targets ERK Pathway‐Driven Cancers: A Therapeutic Approach Toward Precision Medicine,” Molecular Cancer Therapeutics 19, no. 2 (2020): 325–336, 10.1158/1535-7163.MCT-19-0183. 31744895

[121] Y. Y. Sun , H. Zhang , R. R. Ma , et al., “Long Non‐Coding RNA AK025387 Promotes Cell Migration and Invasion of Gastric Cancer,” Frontiers in Oncology 10 (2020): 633, 10.3389/fonc.2020.00633. 32509569 PMC7251172

[122] X. Tian , Y. Zhao , Z. Yang , Q. Lu , L. Zhou , and S. Zheng , “USP15 Regulates p66Shc Stability Associated With Drp1 Activation in Liver Ischemia/Reperfusion,” Cell Death & Disease 13, no. 9 (2022): 823, 10.1038/s41419-022-05277-8. 36163170 PMC9512921

[123] Z. Wang , Y. Zhao , H. Zhao , et al., “Inhibition of p66Shc Oxidative Signaling via CA‐Induced Upregulation of miR‐203a‐3p Alleviates Liver Fibrosis Progression,” Molecular Therapy—Nucleic Acids 21 (2020): 751–763, 10.1016/j.omtn.2020.07.013. 32781430 PMC7417942

[124] Z. Wang , Y. Zhao , R. Sun , et al., “Circ‐CBFB Upregulates p66Shc to Perturb Mitochondrial Dynamics in APAP‐Induced Liver Injury,” Cell Death & Disease 11, no. 11 (2020): 953, 10.1038/s41419-020-03160-y. 33159035 PMC7648761

[125] N. Shin , H. J. Shin , Y. Yi , et al., “p66shc siRNA‐Encapsulated PLGA Nanoparticles Ameliorate Neuropathic Pain Following Spinal Nerve Ligation,” Polymers (Basel) 12, no. 5 (2020): 1014, 10.3390/polym12051014. 32365512 PMC7284875

[126] Y. Zhang , C. Wang , J. Lu , et al., “Targeting of miR‐96‐5p by Catalpol Ameliorates Oxidative Stress and Hepatic Steatosis in LDLr−/− Mice via p66shc/Cytochrome C Cascade,” Aging (Albany NY) 12, no. 3 (2020): 2049–2069, 10.18632/aging.102721. 32023549 PMC7041734

[127] Y. Song , H. Yu , Q. Sun , et al., “Grape Seed Proanthocyanidin Extract Targets p66Shc to Regulate Mitochondrial Biogenesis and Dynamics in Diabetic Kidney Disease,” Frontiers in Pharmacology 13 (2022): 1035755, 10.3389/fphar.2022.1035755. 36686673 PMC9853208

[128] C. H. Lai , K. Xu , J. Zhou , et al., “DEPDC1B Is a Tumor Promotor in Development of Bladder Cancer Through Targeting SHC1,” Cell Death & Disease 11, no. 11 (2020): 986, 10.1038/s41419-020-03190-6. 33203836 PMC7672062

[129] A. Onnis , C. Cassioli , F. Finetti , and C. T. Baldari , “Regulation of Selective B Cell Autophagy by the pro‐Oxidant Adaptor p66SHC,” Frontiers in Cell and Developmental Biology 8 (2020): 193, 10.3389/fcell.2020.00193. 32274384 PMC7113388

[130] D. D. Cave , M. Di Guida , V. Costa , et al., “TGF‐beta1 Secreted by Pancreatic Stellate Cells Promotes Stemness and Tumourigenicity in Pancreatic Cancer Cells Through L1CAM Downregulation,” Oncogene 39, no. 21 (2020): 4271–4285, 10.1038/s41388-020-1289-1. 32291413 PMC7239770

[131] T. T. Huang , W. J. Sun , H. Y. Liu , H. L. Ma , and B. X. Cui , “p66Shc‐Mediated Oxidative Stress Is Involved in Gestational Diabetes Mellitus,” World Journal of Diabetes 12, no. 11 (2021): 1894–1907, 10.4239/wjd.v12.i11.1894. 34888014 PMC8613666

[132] M. Gierhardt , O. Pak , A. Sydykov , et al., “Genetic Deletion of p66shc and/or Cyclophilin D Results in Decreased Pulmonary Vascular Tone,” Cardiovascular Research 118, no. 1 (2022): 305–315, 10.1093/cvr/cvaa310. 33119054 PMC8752355

[133] L. Feng , S. Wang , F. Chen , et al., “Hepatic Knockdown of Endothelin Type A Receptor (ETAR) Ameliorates Hepatic Insulin Resistance and Hyperglycemia Through Suppressing p66Shc‐Mediated Mitochondrial Fragmentation in High‐Fat Diet‐Fed Mice,” Diabetes, Metabolic Syndrome and Obesity: Targets and Therapy 14 (2021): 963–981, 10.2147/DMSO.S299570. 33688230 PMC7936928

[134] D. Wang , T. Wang , R. Wang , et al., “Suppression of p66Shc Prevents Hyperandrogenism‐Induced Ovarian Oxidative Stress and Fibrosis,” Journal of Translational Medicine 18, no. 1 (2020): 84, 10.1186/s12967-020-02249-4. 32066482 PMC7027222

[135] C. Fu , M. Yuan , J. Sun , et al., “RNA‐Binding Motif Protein 11 (RBM11) Serves as a Prognostic Biomarker and Promotes Ovarian Cancer Progression,” Disease Markers 2021 (2021): 3037337, 10.1155/2021/3037337. 34434291 PMC8382552

[136] Y. Jiang , L. Xu , X. Zhu , X. Zhu , X. Xu , and J. Li , “Hyperglycemic Stress Induces Oxidative Damage of Enteric Glial Cells by Triggering Redoxosomes/p66SHC Activation,” Redox Report 29, no. 1 (2024): 2324234, 10.1080/13510002.2024.2324234. 38444386 PMC10919305

[137] C. C. Tsai , Y. S. H. Yang , Y. F. Chen , et al., “Integrins and Actions of Androgen in Breast Cancer,” Cells 12, no. 17 (2023): 2126, 10.3390/cells12172126. 37681860 PMC10486718

[138] W. Huang , L. J. Hickson , A. Eirin , J. L. Kirkland , and L. O. Lerman , “Cellular Senescence: The Good, the Bad and the Unknown,” Nature Reviews Nephrology 18, no. 10 (2022): 611–627, 10.1038/s41581-022-00601-z. 35922662 PMC9362342

[139] C. Ren , X. Zhou , X. Bao , et al., “Dioscorea Zingiberensis Ameliorates Diabetic Nephropathy by Inhibiting NLRP3 Inflammasome and Curbing the Expression of p66Shc in High‐Fat Diet/Streptozotocin‐Induced Diabetic Mice,” Journal of Pharmacy and Pharmacology 73, no. 9 (2021): 1218–1229, 10.1093/jpp/rgab053. 34061184

[140] S. Rossi , V. Tatangelo , M. Dichiara , et al., “A Novel Potent Class I HDAC Inhibitor Reverses the STAT4/p66Shc Apoptotic Defect in B Cells From Chronic Lymphocytic Leukemia Patients,” Biomedicine & Pharmacotherapy 174 (2024): 116537, 10.1016/j.biopha.2024.116537. 38579402

[141] J. Abe and B. C. Berk , “Hypoxia and HIF‐1α Stability: Another Stress‐Sensing Mechanism for Shc,” Circulation Research 91, no. 1 (2002): 4–6, 10.1161/01.res.0000026654.65882.55. 12114314

[142] A. Fukushi , H.‐D. Kim , Y.‐C. Chang , and C.‐H. Kim , “Revisited Metabolic Control and Reprogramming Cancers by Means of the Warburg Effect in Tumor Cells,” International Journal of Molecular Sciences 23, no. 17 (2022): 10037, 10.3390/ijms231710037. 36077431 PMC9456516

[143] X. Liu , J. Zhang , T. Yi , et al., “Decoding Tumor Angiogenesis: Pathways, Mechanisms, and Future Directions in Anti‐Cancer Strategies,” Biomarker Research 13, no. 1 (2025): 62, 10.1186/s40364-025-00779-x. 40251641 PMC12007322

[144] E. C. Cañedo , S. Totten , R. Ahn , et al., “p66ShcA Potentiates the Cytotoxic Response of Triple‐Negative Breast Cancers to PARP Inhibitors,” JCI Insight 6, no. 4 (2021): e138382, 10.1172/jci.insight.138382. 33470989 PMC7934920

[145] K. D. Wright , B. S. Miller , S. El‐Meanawy , et al., “The p52 Isoform of SHC1 Is a Key Driver of Breast Cancer Initiation,” Breast Cancer Research 21, no. 1 (2019): 74, 10.1186/s13058-019-1155-7. 31202267 PMC6570928

[146] T. Okamura , Y. Hashimoto , M. Hamaguchi , A. Obora , T. Kojima , and M. Fukui , “Triglyceride‐Glucose Index (TyG Index) is a Predictor of Incident Colorectal Cancer: A Population‐Based Longitudinal Study,” BMC Endocrine Disorders 20, no. 1 (2020): 113, 10.1186/s12902-020-00581-w. 32709256 PMC7379831

[147] L. J. Jin , W. B. Chen , X. Y. Zhang , J. Bai , H. C. Zhao , and Z. Y. Wang , “Analysis of Factors Potentially Predicting Prognosis of Colorectal Cancer,” World Journal of Gastrointestinal Oncology 11, no. 12 (2019): 1206–1217, 10.4251/wjgo.v11.i12.1206. 31908725 PMC6937433

[148] X. Li , Z. Xu , W. Du , et al., “Aiolos Promotes Anchorage Independence by Silencing p66Shc Transcription in Cancer Cells,” Cancer Cell 25, no. 5 (2014): 575–589, 10.1016/j.ccr.2014.03.020. 24823637 PMC4070880

[149] A. Lone , R. A. Harris , O. Singh , D. H. Betts , and R. C. Cumming , “p66Shc Activation Promotes Increased Oxidative Phosphorylation and Renders CNS Cells More Vulnerable to Amyloid Beta Toxicity,” Scientific Reports 8, no. 1 (2018): 17081, 10.1038/s41598-018-35114-y. 30459314 PMC6244282

[150] E. R. Galimov , B. V. Chernyak , A. S. Sidorenko , A. V. Tereshkova , and P. M. Chumakov , “Prooxidant Properties of p66shc Are Mediated by Mitochondria in Human Cells,” PLoS One 9, no. 3 (2014): e86521, 10.1371/journal.pone.0086521. 24618848 PMC3950296

[151] P. T. Campbell , C. C. Newton , A. V. Patel , E. J. Jacobs , and S. M. Gapstur , “Diabetes and Cause‐Specific Mortality in a Prospective Cohort of One Million U.S. Adults,” Diabetes Care 35, no. 9 (2012): 1835–1844, 10.2337/dc12-0002. 22699290 PMC3425000

[152] N. Capitani , O. M. Lucherini , E. Sozzi , et al., “Impaired Expression of p66Shc, a Novel Regulator of B‐Cell Survival, in Chronic Lymphocytic Leukemia,” Blood 115, no. 18 (2010): 3726–3736, 10.1182/blood-2009-08-239244. 20061561

[153] L. Patrussi , N. Capitani , C. Ulivieri , et al., “p66Shc Deficiency in the Eμ‐TCL1 Mouse Model of Chronic Lymphocytic Leukemia Enhances Leukemogenesis by Altering the Chemokine Receptor Landscape,” Haematologica 104, no. 10 (2019): 2040–2052, 10.3324/haematol.2018.209981. 30819907 PMC6886430

[154] N. Hamdani , S. Costantino , A. Mügge , et al., “Leveraging Clinical Epigenetics in Heart Failure With Preserved Ejection Fraction: A Call for Individualized Therapies,” European Heart Journal 42, no. 20 (2021): 1940–1958. 10.1093/eurheartj/ehab197 PMC8921660 33948637

[155] L. Haronikova , O. Bonczek , P. Zatloukalova , et al., “Resistance Mechanisms to Inhibitors of p53‐MDM2 Interactions in Cancer Therapy: Can We Overcome Them?,” Cellular & Molecular Biology Letters 26, no. 1 (2021): 53, 10.1186/s11658-021-00293-6. 34911439 PMC8903693

[156] C. D. Willey , D. Xiao , T. Tu , et al., “Enzastaurin (LY317615), a Protein Kinase C Beta Selective Inhibitor, Enhances Antiangiogenic Effect of Radiation,” International Journal of Radiation Oncology, Biology, Physics 77, no. 5 (2010): 1518–1526, 10.1016/j.ijrobp.2009.06.044. 19906497 PMC3688843

[157] L. Rawat , H. Hegde , S. L. Hoti , and V. Nayak , “Piperlongumine Induces ROS Mediated Cell Death and Synergizes Paclitaxel in Human Intestinal Cancer Cells,” Biomedicine & Pharmacotherapy 128 (2020): 110243, 10.1016/j.biopha.2020.110243. 32470748

[158] H. G. Khalefa , M. A. Shawki , R. Aboelhassan , and L. M. El Wakeel , “Evaluation of the Effect of N‐Acetylcysteine on the Prevention and Amelioration of Paclitaxel‐Induced Peripheral Neuropathy in Breast Cancer Patients: A Randomized Controlled Study,” Breast Cancer Research and Treatment 183, no. 1 (2020): 117–125, 10.1007/s10549-020-05762-8. 32601973

[159] L. Lopresti , V. Tatangelo , C. T. Baldari , and L. Patrussi , “Rewiring the T Cell‐Suppressive Cytokine Landscape of the Tumor Microenvironment: A New Frontier for Precision Anti‐Cancer Therapy,” Frontiers in Immunology 15 (2024): 1418527, 10.3389/fimmu.2024.1418527. 39281678 PMC11392891

[160] F. Crea , “Focus on Translational Vascular Biology: New Therapeutic Targets in Hypertension, Aortic Aneurysm, and Atherosclerosis,” European Heart Journal 44, no. 29 (2023): 2645–2649, 10.1093/eurheartj/ehad481. 37527405

[161] R. K. Singh , S. Kumar , S. Kumar , et al., “Potential Implications of Protein Kinase Cα in Pathophysiological Conditions and Therapeutic Interventions,” Life Sciences 330 (2023): 121999, 10.1016/j.lfs.2023.121999. 37536614

[162] S. Cotino‐Nájera , L. A. Herrera , G. Domínguez‐Gómez , and J. Díaz‐Chávez , “Molecular Mechanisms of Resveratrol as Chemo and Radiosensitizer in Cancer,” Frontiers in Pharmacology 14 (2023): 1287505, 10.3389/fphar.2023.1287505. 38026933 PMC10667487

[163] R. A. Zinovkin , K. G. Lyamzaev , and B. V. Chernyak , “Current Perspectives of Mitochondria‐Targeted Antioxidants in Cancer Prevention and Treatment,” Frontiers in Cell and Developmental Biology 11 (2023): 1048177, 10.3389/fcell.2023.1048177. 37009472 PMC10060896

[164] M. M. Gomari , S. Abkhiz , T. G. Pour , et al., “Peptidomimetics in Cancer Targeting,” Molecular Medicine 28, no. 1 (2022): 146, 10.1186/s10020-022-00577-3. 36476230 PMC9730693

[165] L. Patrussi , N. Capitani , and C. T. Baldari , “P66Shc: A Pleiotropic Regulator of B Cell Trafficking and a Gatekeeper in Chronic Lymphocytic Leukemia,” Cancers (Basel) 12, no. 4 (2020): 1006, 10.3390/cancers12041006. 32325830 PMC7226591

[166] L. Patrussi , N. Capitani , and C. T. Baldari , “Interleukin (IL)‐9 Supports the Tumor‐Promoting Environment of Chronic Lymphocytic Leukemia,” Cancers 13, no. 24 (2021): 6301, 10.3390/cancers13246301. 34944921 PMC8699356

[167] M. A. Ingersoll , D. R. Miller , O. Martinez , et al., “Statin Derivatives as Therapeutic Agents for Castration‐Resistant Prostate Cancer,” Cancer Letters 383, no. 1 (2016): 94–105, 10.1016/j.canlet.2016.09.008. 27687622 PMC5086260

[168] D. R. Miller , M. A. Ingersoll , A. Chatterjee , et al., “p66Shc Protein Through a Redox Mechanism Enhances the Progression of Prostate Cancer Cells Towards Castration‐Resistance,” Free Radical Biology & Medicine 139 (2019): 24–34, 10.1016/j.freeradbiomed.2019.05.015. 31100478 PMC6620027

[169] H. Kanda and K. Yamawaki , “Bardoxolone Methyl: Drug Development for Diabetic Kidney Disease,” Clinical and Experimental Nephrology 24, no. 10 (2020): 857–864, 10.1007/s10157-020-01917-5. 32594372 PMC7497696

[170] Q. Li and L. Wang , “Navigating the Complex Role of Senescence in Liver Disease,” Chinese Medical Journal 137, no. 24 (2024): 3061–3072, 10.1097/CM9.0000000000003439. 39679454 PMC11706581

[171] J. N. Zhu , Y. H. Fu , Z. Q. Hu , et al., “Activation of miR‐34a‐5p/Sirt1/p66shc Pathway Contributes to Doxorubicin‐Induced Cardiotoxicity,” Scientific Reports 7, no. 1 (2017): 11879, 10.1038/s41598-017-12192-y. 28928469 PMC5605522

[172] W. Shan , L. Gao , W. Zeng , et al., “Activation of the SIRT1/p66shc Antiapoptosis Pathway via Carnosic Acid‐Induced Inhibition of miR‐34a Protects Rats Against Nonalcoholic Fatty Liver Disease,” Cell Death & Disease 6, no. 7 (2015): e1833, 10.1038/cddis.2015.196. 26203862 PMC4650741

[173] M. Zhang , J. Tang , H. Shan , et al., “p66Shc Mediates Mitochondrial Dysfunction Dependent on PKC Activation in Airway Epithelial Cells Induced by Cigarette Smoke,” Oxidative Medicine and Cellular Longevity 2018 (2018): 5837123, 10.1155/2018/5837123. 29849902 PMC5925171

[174] Y. Dong , J. Peng , X. Zhang , Q. Wang , and X. Lyu , “SAHA Inhibits Lung Fibroblast Activation by Increasing p66Shc Expression Epigenetically,” Aging Medicine 7, no. 6 (2024): 790–801, 10.1002/agm2.12385. 39777101 PMC11702475

[175] S. Ciciliot , M. Albiero , L. Menegazzo , et al., “p66Shc Deletion or Deficiency Protects From Obesity but Not Metabolic Dysfunction in Mice and Humans,” Diabetologia 58, no. 10 (2015): 2352–2360, 10.1007/s00125-015-3667-8. 26122877

[176] M. K. Chamgordani , A. Bardestani , S. Ebrahimpour , and A. Esmaeili , “In Diabetic Male Wistar Rats, Quercetin‐Conjugated Superparamagnetic Iron Oxide Nanoparticles Have an Effect on the SIRT1/p66Shc‐Mediated Pathway Related to Cognitive Impairment,” BMC Pharmacology and Toxicology 24, no. 1 (2023): 81, 10.1186/s40360-023-00725-3. 38129872 PMC10734159

[177] X. Yang , R. Xu , Y. Lin , et al., “Recombinant Adenovirus of Human p66Shc Inhibits MCF‐7 Cell Proliferation,” Scientific Reports 6 (2016): 31534, 10.1038/srep31534. 27530145 PMC4987618

[178] C. Zhang , W. B. Dong , S. Zhao , et al., “Construction of p66Shc Gene Interfering Lentivirus Vectors and Its Effects on Alveolar Epithelial Cells Apoptosis Induced by Hyperoxia,” Drug Design, Development and Therapy 10 (2016): 2611–2622, 10.2147/DDDT.S84820. 27574400 PMC4993261

[179] S. Chen , S. N. Heendeniya , B. T. Le , et al., “Splice‐Modulating Antisense Oligonucleotides as Therapeutics for Inherited Metabolic Diseases,” BioDrugs 38, no. 2 (2024): 177–203, 10.1007/s40259-024-00644-7. 38252341 PMC10912209

[180] L. S. Terada , “Shc and the Mechanotransduction of Cellular Anchorage and Metastasis,” Small GTPases 10, no. 1 (2019): 64–71, 10.1080/21541248.2016.1273172. 28632027 PMC6343529

[181] F. R. Prandi , D. Lecis , F. Illuminato , et al., “Epigenetic Modifications and Non‐Coding RNA in Diabetes‐Mellitus‐Induced Coronary Artery Disease: Pathophysiological Link and New Therapeutic Frontiers,” International Journal of Molecular Sciences 23, no. 9 (2022): 4589, 10.3390/ijms23094589. 35562979 PMC9105558

[182] A. A. Balachandran , P. Raguraman , K. Rahimizadeh , and R. N. Veedu , “Splice‐Switching Antisense Oligonucleotides Targeting Extra‐And Intracellular Domains of Epidermal Growth Factor Receptor in Cancer Cells,” Biomedicine 11, no. 12 (2023): 3299, 10.3390/biomedicines11123299. PMC10741442 38137520

[183] H. Dai , R. Abdullah , X. Wu , et al., “Pancreatic Cancer: Nucleic Acid Drug Discovery and Targeted Therapy,” Frontiers in Cell and Developmental Biology 10 (2022): 855474, 10.3389/fcell.2022.855474. 35652096 PMC9149368

[184] Y. Li , S. Chen , K. Rahimizadeh , Z. Zhang , and R. N. Veedu , “Inhibition of Survivin by 2'‐O‐Methyl Phosphorothioate‐Modified Steric‐Blocking Antisense Oligonucleotides,” RSC Advances 14, no. 19 (2024): 13336–13341, 10.1039/d4ra01925c. 38660533 PMC11040434

[185] T. W. Madanayake , E. A. Welsh , L. N. F. Darville , et al., “Inhibition of Epidermal Growth Factor Receptor Signaling by Antisense Oligonucleotides as a Novel Approach to Epidermal Growth Factor Receptor Inhibition,” Nucleic Acid Therapeutics 32, no. 5 (2022): 391–400, 10.1089/nat.2021.0101. 35861718 PMC9595651

[186] E. Çakan , O. D. Lara , A. Szymanowska , et al., “Therapeutic Antisense Oligonucleotides in Oncology: From Bench to Bedside,” Cancers (Basel) 16, no. 17 (2024): 2940, 10.3390/cancers16172940. 39272802 PMC11394571

[187] B. Cetin Ersen , B. Goncu , A. Dag , and G. Birlik Demirel , “GLUT‐Targeting Phototherapeutic Nanoparticles for Synergistic Triple Combination Cancer Therapy,” ACS Applied Materials & Interfaces 15 (2023): 9080–9098, 10.1021/acsami.2c21180. 36780418

[188] M. A. De Velasco , Y. Kura , K. Sakai , et al., “Targeting Castration‐Resistant Prostate Cancer With Androgen Receptor Antisense Oligonucleotide Therapy,” JCI Insight 4, no. 17 (2019): e122688, 10.1172/jci.insight.122688. 31484823 PMC6777919

[189] K. Dhuri , C. Bechtold , E. Quijano , et al., “Antisense Oligonucleotides: An Emerging Area in Drug Discovery and Development,” Journal of Clinical Medicine 9, no. 6 (2020): 2004, 10.3390/jcm9062004. 32604776 PMC7355792

[190] A. Denker , “Antisense Oligonucleotides as Therapeutics for Difficult‐To‐Drug Targets in Oncology,” in Proceedings of the AACR Special Conference in Cancer Research: RNAs as Drivers, Targets, and Therapeutics in Cancer (AACR, Molecular Cancer Therapeutics, 2024), 10.1158/1538-8514.Rnadrivers24-i008.

[191] Y. Yan , H. Li , H. Yao , and X. Cheng , “Nanodelivery Systems Delivering Hypoxia‐Inducible Factor‐1 Alpha Short Interfering RNA and Antisense Oligonucleotide for Cancer Treatment,” Frontiers in Nanotechnology 4 (2022): 932976, 10.3389/fnano.2022.932976.

[192] C. Dong , J. Wu , Y. Chen , J. Nie , and C. Chen , “Activation of PI3K/AKT/mTOR Pathway Causes Drug Resistance in Breast Cancer,” Frontiers in Pharmacology 12 (2021): 628690, 10.3389/fphar.2021.628690. 33790792 PMC8005514

[193] D. A. Sabbah , R. Hajjo , S. K. Bardaweel , and H. A. Zhong , “Targeting the PI3K/AKT Signaling Pathway in Anticancer Research: A Recent Update on Inhibitor Design and Clinical Trials (2020–2023),” Expert Opinion on Therapeutic Patents 34, no. 3 (2024): 141–158, 10.1080/13543776.2024.2338100. 38557273

[194] R. Liu , L. Peng , L. Zhou , Z. Huang , C. Zhou , and C. Huang , “Oxidative Stress in Cancer Immunotherapy: Molecular Mechanisms and Potential Applications,” Antioxidants 11, no. 5 (2022): 853, 10.3390/antiox11050853. 35624717 PMC9137834

[195] N. Goel , M. E. Foxall , C. B. Scalise , J. A. Wall , and R. C. Arend , “Strategies in Overcoming Homologous Recombination Proficiency and PARP Inhibitor Resistance,” Molecular Cancer Therapeutics 20, no. 9 (2021): 1542–1549, 10.1158/1535-7163.MCT-20-0992. 34172532 PMC9066363

[196] Y. Li , S. Gao , X. Du , J. Ji , Y. Xi , and G. Zhai , “Advances in Autophagy as a Target in the Treatment of Tumours,” Journal of Drug Targeting 30, no. 2 (2022): 166–187, 10.1080/1061186X.2021.1961792. 34319838

[197] M. Yu , J. Chen , Z. Xu , et al., “Development and Safety of PI3K Inhibitors in Cancer,” Archives of Toxicology 97, no. 3 (2023): 635–650, 10.1007/s00204-023-03440-4. 36773078 PMC9968701

[198] M. Nakazawa , C. Paller , and N. Kyprianou , “Mechanisms of Therapeutic Resistance in Prostate Cancer,” Current Oncology Reports 19, no. 2 (2017): 13, 10.1007/s11912-017-0568-7. 28229393 PMC5812366

[199] J. Taugner , L. Käsmann , C. Eze , et al., “Durvalumab After Chemoradiotherapy for PD‐L1 Expressing Inoperable Stage III NSCLC Leads to Significant Improvement of Local‐Regional Control and Overall Survival in the Real‐World Setting,” Cancers (Basel) 13, no. 7 (2021): 1613, 10.3390/cancers13071613. 33807324 PMC8037429

[200] D. Zhou , X. Zhu , and Y. Xiao , “CAR‐T Cell Combination Therapies in Hematologic Malignancies,” Experimental Hematology & Oncology 13, no. 1 (2024): 69, 10.1186/s40164-024-00536-0. 39026380 PMC11264744

[201] Y. Guo , Y. Jin , B. Qu , Y. Zhang , J. Che , and X. Dong , “An Updated Patent Review of Akt Inhibitors (2016‐Present),” Expert Opinion on Therapeutic Patents 31, no. 9 (2021): 837–849, 10.1080/13543776.2021.1915291. 33834942

[202] N. E. Uko , O. F. Guner , D. F. Matesic , and J. P. Bowen , “Akt Pathway Inhibitors,” Current Topics in Medicinal Chemistry 20, no. 10 (2020): 883–900, 10.2174/1568026620666200224101808. 32091335

[203] S. Guo , S. Zheng , M. Liu , and G. Wang , “Novel Anti‐Cancer Stem Cell Compounds: A Comprehensive Review,” Pharmaceutics 16, no. 8 (2024): 1024, 10.3390/pharmaceutics16081024. 39204369 PMC11360402

[204] Z. Zhang , L. Zhou , N. Xie , et al., “Overcoming Cancer Therapeutic Bottleneck by Drug Repurposing,” Signal Transduction and Targeted Therapy 5, no. 1 (2020): 113, 10.1038/s41392-020-00213-8. 32616710 PMC7331117

[205] S. Veeramani , Y. W. Chou , F. C. Lin , et al., “Reactive Oxygen Species Induced by p66Shc Longevity Protein Mediate Nongenomic Androgen Action via Tyrosine Phosphorylation Signaling to Enhance Tumorigenicity of Prostate Cancer Cells,” Free Radical Biology & Medicine 53, no. 1 (2012): 95–108, 10.1016/j.freeradbiomed.2012.03.024. 22561705 PMC3384717

[206] D. Xiao and S. V. Singh , “p66Shc Is Indispensable for Phenethyl Isothiocyanate‐Induced Apoptosis in Human Prostate Cancer Cells,” Cancer Research 70, no. 8 (2010): 3150–3158, 10.1158/0008-5472.CAN-09-4451. 20354186 PMC2855757

[207] G. Liu , B. Xie , L. Gong , J. Zhou , and G. Shu , “The Expression of p66Shc Protein in Benign, Premalignant, and Malignant Gastrointestinal Lesions,” Pathology Oncology Research 20, no. 3 (2014): 733–739, 10.1007/s12253-014-9754-1. 24599562

[208] D. H. Betts , N. T. Bain , and P. Madan , “The p66(Shc) Adaptor Protein Controls Oxidative Stress Response in Early Bovine Embryos,” PLoS One 9, no. 1 (2014): e86978, 10.1371/journal.pone.0086978. 24475205 PMC3901717

[209] Z. Ma , Z. Liu , R. F. Wu , and L. S. Terada , “p66(Shc) Restrains Ras Hyperactivation and Suppresses Metastatic Behavior,” Oncogene 29, no. 41 (2010): 5559–5567, 10.1038/onc.2010.326. 20676142 PMC3045677

[210] M. R. Wieckowski , C. M. Deus , R. Couto , et al., “Measuring p66Shc Signaling Pathway Activation and Mitochondrial Translocation in Cultured Cells,” Current Protocols in Toxicology 66 (2015): 25.6.1–25.6.21, 10.1002/0471140856.tx2506s66. 26523473

[211] P. C. Havugimana , G. T. Hart , T. Nepusz , et al., “A Census of Human Soluble Protein Complexes,” Cell 150, no. 5 (2012): 1068–1081, 10.1016/j.cell.2012.08.011. 22939629 PMC3477804

[212] F. Cattaneo , L. Patrussi , N. Capitani , et al., “Expression of the p66Shc Protein Adaptor Is Regulated by the Activator of Transcription STAT4 in Normal and Chronic Lymphocytic Leukemia B Cells,” Oncotarget 7, no. 35 (2016): 57086–57098, 10.18632/oncotarget.10977. 27494881 PMC5302975

[213] R. Wang , Q. Sun , P. Wang , et al., “Notch and Wnt/Beta‐Catenin Signaling Pathway Play Important Roles in Activating Liver Cancer Stem Cells,” Oncotarget 7, no. 5 (2016): 5754–5768, 10.18632/oncotarget.6805. 26735577 PMC4868719

[214] P. Sansone , G. Storci , C. Giovannini , et al., “p66Shc/Notch‐3 Interplay Controls Self‐Renewal and Hypoxia Survival in Human Stem/Progenitor Cells of the Mammary Gland Expanded In Vitro as Mammospheres,” Stem Cells 25, no. 3 (2007): 807–815, 10.1634/stemcells.2006-0442. 17158237

[215] N. Kothari , H. Postwala , A. Pandya , A. Shah , Y. Shah , and M. R. Chorawala , “Establishing the Applicability of Cancer Vaccines in Combination With Chemotherapeutic Entities: Current Aspect and Achievable Prospects,” Medical Oncology 40, no. 5 (2023): 135, 10.1007/s12032-023-02003-y. 37014489

[216] V. Thiruchenthooran , M. Espina , M. Switalska , et al., “Combination of Indomethacin With Nanostructured Lipid Carriers for Effective Anticancer Therapy,” International Journal of Nanomedicine 19 (2024): 7033–7048, 10.2147/IJN.S464239. 39015675 PMC11249952

